# Deaths Ascribed to Non-Communicable Diseases among Rural Kenyan Adults Are Proportionately Increasing: Evidence from a Health and Demographic Surveillance System, 2003–2010

**DOI:** 10.1371/journal.pone.0114010

**Published:** 2014-11-26

**Authors:** Penelope A. Phillips-Howard, Kayla F. Laserson, Nyaguara Amek, Caryl M. Beynon, Sonia Y. Angell, Sammy Khagayi, Peter Byass, Mary J. Hamel, Anne M. van Eijk, Emily Zielinski-Gutierrez, Laurence Slutsker, Kevin M. De Cock, John Vulule, Frank O. Odhiambo

**Affiliations:** 1 Department of Clinical Sciences, Liverpool School of Tropical Medicine, Liverpool, United Kingdom; 2 Centre for Global Health Research, Kenya Medical Research Institute, Kisumu, Kenya; 3 Center for Global Health, Centers for Disease Control and Prevention, Atlanta, GA, United States of America; 4 Center for Public Health, Liverpool John Moores University, Liverpool, United Kingdom; 5 Department of Non-Communicable Diseases, Division of Global Health Protection, Centers for Disease Control and Prevention, Atlanta, GA, United States of America; 6 Umeå Centre for Global Health Research, Umeå University, Umeå, Sweden; 7 Division of Parasitic Diseases and Malaria, Centers for Disease Control and Prevention, Atlanta, GA, United States of America; 8 Division of Global HIV and AIDS, Centers for Disease Control and Prevention, Kisumu, Kenya; 9 Center for Global Health, Centers for Disease Control and Prevention-Kenya, Nairobi, Kenya; University of Massachusetts Medical School, United States of America

## Abstract

**Background:**

Non-communicable diseases (NCDs) result in more deaths globally than other causes. Monitoring systems require strengthening to attribute the NCD burden and deaths in low and middle-income countries (LMICs). Data from health and demographic surveillance systems (HDSS) can contribute towards this goal.

**Methods and Findings:**

Between 2003 and 2010, 15,228 deaths in adults aged 15 years (y) and older were identified retrospectively using the HDSS census and verbal autopsy in rural western Kenya, attributed into broad categories using InterVA-4 computer algorithms; 37% were ascribed to NCDs, 60% to communicable diseases (CDs), 3% to injuries, and <1% maternal causes. Median age at death for NCDs was 66y and 71y for females and males, respectively, with 43% (39% male, 48% female) of NCD deaths occurring prematurely among adults aged below 65y. NCD deaths were mainly attributed to cancers (35%) and cardio-vascular diseases (CVDs; 29%). The proportionate mortality from NCDs rose from 35% in 2003 to 45% in 2010 (χ^2^ linear trend 93.4; p<0.001). While overall annual mortality rates (MRs) for NCDs fell, cancer-specific MRs rose from 200 to 262 per 100,000 population, mainly due to increasing deaths in adults aged 65y and older, and to respiratory neoplasms in all age groups. The substantial fall in CD MRs resulted in similar MRs for CDs and NCDs among all adult females by 2010. NCD MRs for adults aged 15y to <65y fell from 409 to 183 per 100,000 among females and from 517 to 283 per 100,000 population among males. NCD MRs were higher among males than females aged both below, and at or above, 65y.

**Conclusions:**

NCDs constitute a significant proportion of deaths in rural western Kenya. Evidence of the increasing contribution of NCDs to overall mortality supports international recommendations to introduce or enhance prevention, screening, diagnosis and treatment programmes in LMICs.

## Introduction

Non-communicable diseases (NCDs) are reported to be responsible for two out of every three deaths worldwide [1]; of 36 m deaths associated with NCDs globally, 80% occur in low- and middle-income countries (LMICs) [2]–[4]. NCD deaths are mainly due to cardiovascular diseases (CVDs), cancers, chronic respiratory diseases and diabetes [3], [5], [6]; other major causes of NCD mortality include suicide and injury related to depressive disorders, maternal deaths, and road injuries [7]. Ischaemic heart disease is the leading cause of premature mortality worldwide [1]. Globally the proportion of NCD deaths are predicted to rise from 59% in 2002 to 69% of all deaths by 2030 [7]. The relative increase in the NCDs burden has been classified as a global crisis and a barrier to development goals around poverty reduction, health equity, economic stability, and human security [8]. Recent data suggest health care is restricted to hospital level services only [9], and poor access to appropriate care resulting in low survival [10]. A lack of interventions will result in a cumulative economic loss surpassing US$7 trillion in LMICs between 2011–2025 [11]. However, increases in the proportion of deaths due to NCDs also reflects improved life expectancy, increasing exposure to risk factors of NCDs, both due to increasing longevity and to societal changes, including for example, tobacco promotion [4]. The proportional increase in NCD deaths is also a result of reducing CD burden, reflecting improvements in population health. It is expected that as the disposable income of LMIC rises, risk factors for NCD disorders such as cardiovascular diseases will increase in tandem, while an on-going heavy CDs burden will continue [12]. Interventions are needed to address the growing burden of NCDs in African countries [2], [4], [5], [13]–[15]. Evidence of the cause-specific burden of NCDs is required to plan and fund such interventions, but such data do not exist in many counties [16]–[18], and concerns have been raised on the quality of data available for interpreting trends [19].

Areas in sub-Saharan Africa (SSA) with potential to monitor changing trends in causes of death over time can contribute towards global knowledge on disease burdens of LMICs [5], [6]. A health and demographic surveillance system (HDSS) in western Kenya, established through the Kenyan Medical Research Institute (KEMRI) in collaboration with the US Centers for Disease Control and Prevention (CDC) provides such an opportunity [20]. Longitudinal studies on mortality associated with HIV [21], TB [22], maternal [23], trauma [24], and deaths among children [25], and adolescent and young adults [26], have been characterised. This paper aims to examine NCDs mortality patterns over a similar timespan in order to evaluate the contribution of NCDs to all adult deaths and identify trends for attributed causes.

## Materials and Methods

### Study site and population

The HDSS study site is located in a rural part of Siaya County, in western Kenya [20], [27]. The area consists of 385 villages spread over a 700 km^2^ area along the shores of Lake Victoria, with a population in 2010 of 224,500 adults in aged 15 years (y) and above. The population, mainly subsistence farmers, are almost exclusively members of the Luo ethnic group and traditionally polygynous, have been described in detail elsewhere [20], [28].

### Health and Demographic Surveillance System

The HDSS is a population-based system with GPS locational data, that longitudinally records demographic (births, deaths, pregnancies, and in- and out-migrations) information [20]. Household census among the population takes place tri-annually, in January-March, May-August, and October-December, by field staff who visit all households in the study site.

### Verbal autopsy (VA)

Verbal autopsy is conducted in subpopulations covered by HDSS. All deaths in residents, defined as having resided in the area for at least four consecutive months, are identified by local village reporters through ongoing local monitoring, and are validated during the tri-annual census. At least one month following death, and within four months to reduce recall bias, an interviewer returns to the home and records events surrounding the death, using standardized WHO verbal autopsy (VA) questionnaires endorsed by the INDEPTH Network [20], [29], [30], with spouses or another close relative of the deceased. Resident identification numbers allow linkage of each death with HDSS data. In this paper, cause of death was attributed using the InterVA-4 methodology, a new public-domain probabilistic model for interpreting cause of death from VA data [31], [32]. This methodology attributes cause of death compatible with the International Classification of Diseases 10 (ICD-10) categorised into 62 overall groups through a computer simulated algorithm. ICD-10 codes and the respective VA coding and disease categories are listed in Table S1. Indicators required to run the InterVA-4 Model were extracted from VA data and entered into the model to generate cause of death. The model produces a maximum of three probable causes of death and their corresponding likelihoods. In this paper, analyses focus on primary cause of deaths since only 10% received a secondary and <1% received a tertiary diagnosis. The model has a built-in facility to adjust for the prevalence of malaria and HIV/AIDS. Before running, the model was set high for both the diseases. Previous studies in our surveillance area have reported the prevalence of malaria and HIV/AIDs at 33% and 14% respectively [20], [21], [25].

### Ethical considerations

The HDSS protocol and consent procedures, including surveillance and VA, are approved by the Ethical Review Committee of the Kenyan Medical Research Institute (#1801) and by the Centers for Disease Control and Prevention Institutional Review Board (#3308). Following cultural customs, compound heads provide written informed consent for all compound members to participate in HDSS activities. Individuals can refuse to participate at any time. All HDSS census and VA data are maintained on a secure server accessed by authorized researchers only. Named data are securely stored in a MS-SQL database and only authorized data personnel have access rights. Datasets analysed by scientists are stripped of names to protect identity.

### Analyses

For this evaluation, adults were defined as persons aged 15 years and older. Data were extracted from the HDSS database for all adult deaths in residents, generated from the adult VA questionnaire, between January 2003 and December 2010. Primary cause was derived from aggregated ICD-10 codes generated by the InterVA-4 algorithms [31] (Table S1). Median age of death is presented with interquartile ranges (IQR), for grouped causes of NCD deaths. Analyses are stratified by sex, and into two age groups, using 65 years as the break-point (i.e. below; 15y to <65y, and at or above; ≥65y), to investigate trends in the causes and proportion of premature (aged below 65y) NCD deaths. Descriptive data include deaths in Karemo villages captured 2008–2010, but time trends on the absolute number of deaths and mortality rates 2003–2010 exclude these villages. Mortality rates per 100,000 population for CDs and NCDs, and for main NCD causes (as aggregated through ICD-10 codes; Table S1), were estimated by year and age category using mid-year population-point estimates generated from the HDSS census. The age-sex structure of the adult population was examined per year to clarify if relative proportions changed over time. The highly stable population profile precluded the need to make temporal adjustments to the denominator for analysis of rates.

Key social and demographic characteristics generated from questioning the compound head during HDSS census surveys were examined, to compare differences among deaths from NCD and CD, and by sex. This included marital status (ever married, married at time of death, divorced), education (completed primary, secondary school), and socio-economic status (SES). SES quintiles were based on multiple correspondence analysis (MCA) generated from biennial surveys on wealth indicators, reported elsewhere [20], [26]. In this paper we collapsed the five SES quintiles into two, portraying poorest (lowest two quintiles) and less poor (highest three quintiles).

Analyses were conducted using SPSS for Windows (Release v21.0; IBM, Endicott, NY, USA), and EpiInfo Stat Calc (v7; CDC Atlanta, USA). Chi-squared (χ^2^) test for linear trend (LT) determined the significance of changing rates by sex and age over time (2003 to 2010). Pearson's χ^2^ test was used to determine differences between groups. Mantel Haenszel Relative Risks (RR), with Taylor Series 95% confidence intervals (CI), were used to compare annual mortality rates between sexes. We stratified RR analyses for mortality rates by age groups, sex, and year generating a summary χ^2^, with a Mantel Haenszel weighted RR (MHRR) and Greenlands-Robins 95% CI. Significance was set at 5% or less.

## Results

### Relative contribution of NCD deaths

**Figure 1:**
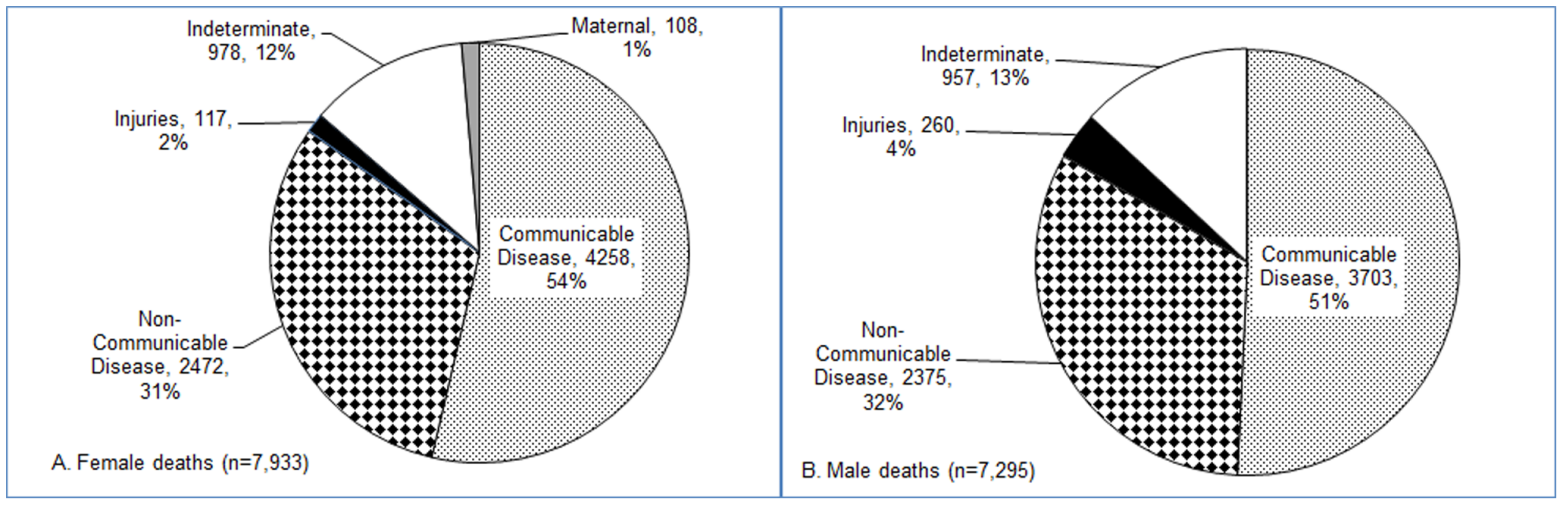
Causes of deaths among all adults aged 15 years and above, rural western Kenya, 2003–2010. A. Female Deaths. B. Male Deaths.

Between 2003 and 2010, 15,228 deaths were recorded in persons living in the HDSS aged 15 years and older. Of these, 13,293 (87%) had a defined primary cause of death, and 1,935 (13%, 957 males, 978 females) were of indeterminate or unknown cause. Figure 1 illustrates primary cause of death for females and males; 52% (54% females, 52% males) were attributed to CDs; 32% (31% females, 33% males) to NCDs, 3% (2% females, 3% males) to injury, <1% (1% females) to maternal causes, and 13% (12% females, 13% males) were indeterminate. Pregnancy- and injury-related deaths are described in detail elsewhere [23], [24].

**Figure 2:**
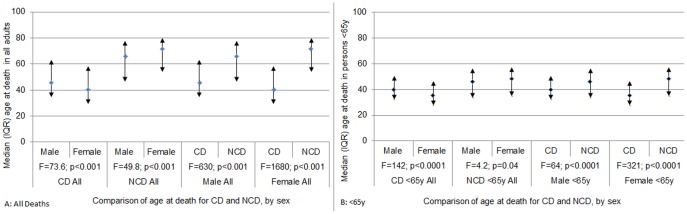
Comparison of median ages at death between CDs and NCDs by sex. A. All Deaths. B. Deaths <65y.

Of the 12,808 adult NCD and CD deaths, two-thirds were 15y to <65y of age, among both female and male deaths. The median age at death among females was significantly lower than males for CD causes, and significantly higher for NCD causes (Figure 2).

**Figure 3:**
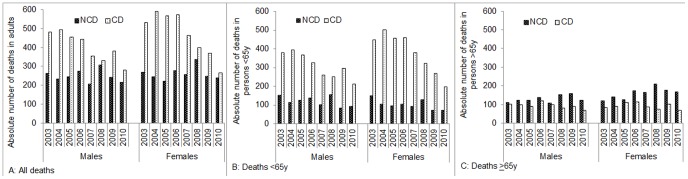
Trends in absolute number of adult deaths attributed to CD and NCD causes in western Kenya, 2003–2010. A. All Deaths. B. Deaths <65y. C. Deaths ≥65y.

Of 11,112 CD and NCD deaths (excluding Karemo) recorded between 2003 and 2010, the overall number of deaths fell by 35% from 1,550 in 2003 to 1,003 in 2010 (Figure 3). While the number of CD deaths dropped 45% (41% males, 49% females) the number of NCD deaths fell 15% (20% males, 10% females). Thus, the proportion of deaths with a primary NCD cause rose over the eight years from 35% to 45% (χ^2^ LT 93.4; p<0.001) of all deaths. This trend was consistent for both sexes (Figure 3). Distribution of deaths stratified by an age threshold of 65y found the proportion of NCD to CD deaths in adults aged <65y varied by year within a range of 20–30%, with a weak trend for an increasing percentage of NCDs over time (Figure 3). Among deaths of persons aged ≥65y there was a significant increase in the percentage of NCD deaths, rising from 55% in 2003 to 68% in 2010 (χ^2^ LT 30.5, p<0.001), evident in both sexes (Figure 3).

### Characteristics of Deaths

Among the 12,808 deaths, there was no difference in marital status between NCD and CD with nearly all (93%) ever married, and half married at the time of death; however, divorce (separation) was almost twice among CD compared with NCD deaths (9% v 17% v 9% RR 1.8; 95% CI 1.6–2.1). Half of persons who had died were in the poorer socio-economic status (SES) two quintiles, with NCD marginally higher (50%) compared with CD (46%). A significantly higher proportion of deaths of persons from CD completed primary school compared with NCD (37% v 19%; p<0.001). Less than 1% of NCD and CD deaths completed secondary schooling. Differences by sex were noted for the proportion in the lowest two SES quintiles with females significantly poorer among NCD compared with CD (59% v 50%, p<0.001), but not among males (NCD and CD, 42%). Differences between NCD and CD for schooling held for both sexes. Married at time of death reversed for males and females; thus while 70% of male NCD deaths were married (compared with 64% of CD), among females 31% of NCD were married and 36% of CD, illustrating the high proportion of female deaths, particularly from NCDs, occurring in widowhood.

### NCDs as primary causes of death

**Figure 4:**
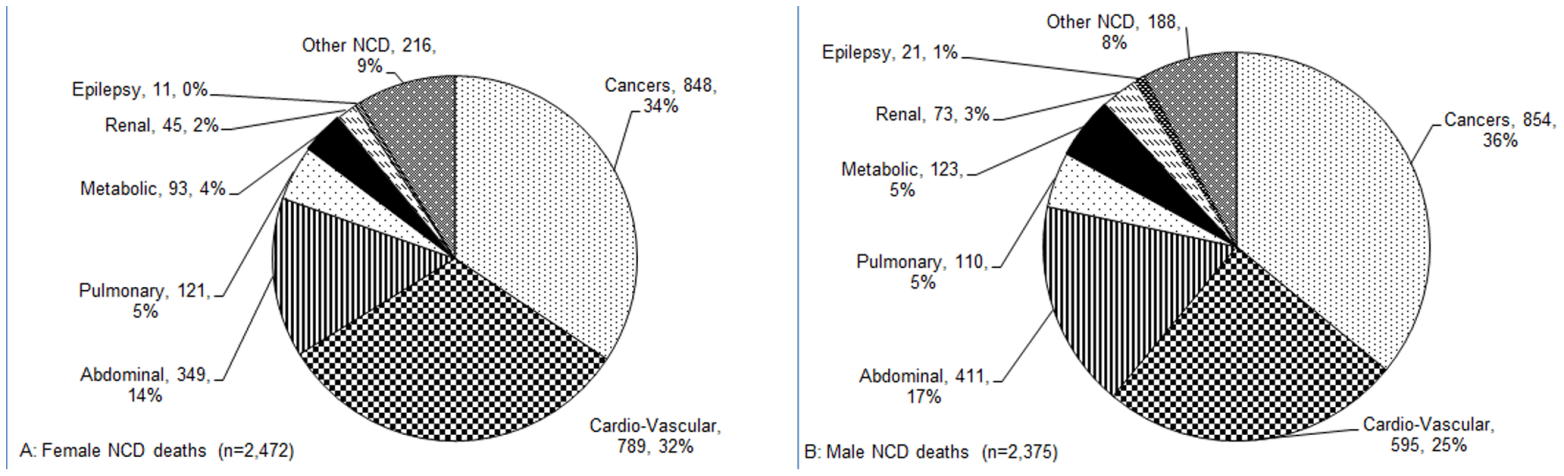
Causes of NCDs deaths among adults in rural western Kenya, 2003 –**2010.** A. Female NCD Deaths. B. Male NCD Deaths.

Among the 4,847 NCD-attributed deaths, two-thirds were ascribed to cancers and CVDs (Figure 4). For males and females combined, cancers were the leading primary cause responsible for 35% of NCD deaths, CVDs were second most common (29%), and abdominal disorders third (16%). Five percent of deaths were each recorded as due to pulmonary, metabolic disorders, renal diseases, and epilepsy, with a further 8% attributed to other NCDs.

Tables S2-S5 list details of 4,107 deaths by study year and thus exclude 740 deaths from Karemo, overall (Table S2, Figure S1), and by type, age threshold, and sex. Figures S1-S9 graphically illustrate these data.

#### Cancers

**Figure 5:**
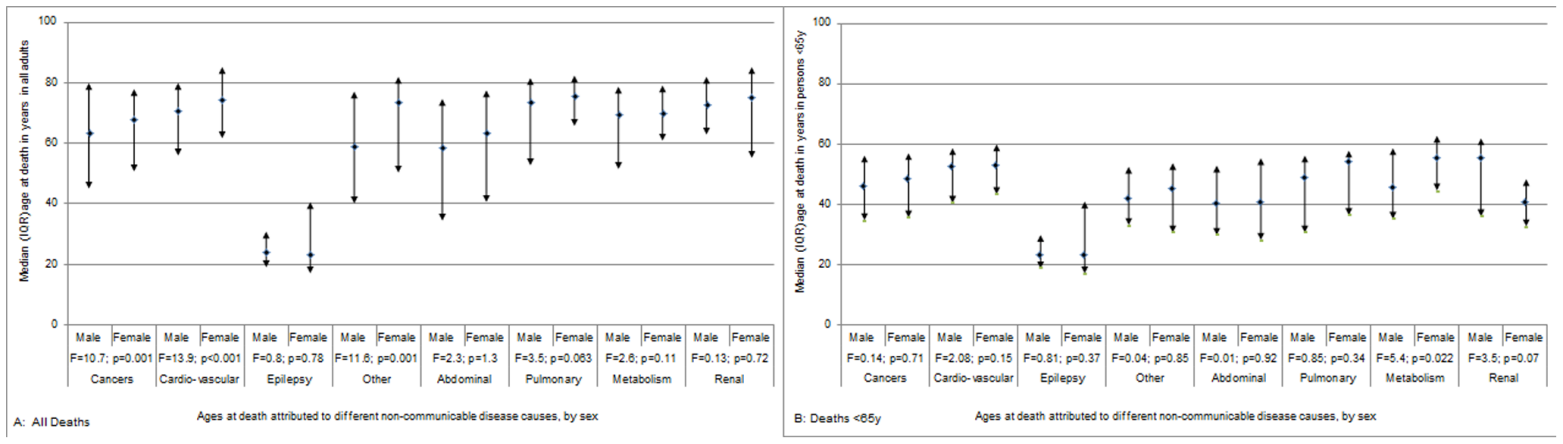
Comparison of median ages of adult deaths by sex, for different NCD causes in rural western Kenya. A. All Deaths. B. Deaths <65y.

Digestive, respiratory/intrathoracic, reproductive, female breast, oral, and other/unspecified (all area total of 1,702 deaths; 854 [50%] male, 848 female). Deaths attributed to neoplasms were distributed equally between males and females. The median age of death for all cancers was 66y (IQR 48-77; Figure 5). Close to half (48%) of deaths were <65y of age. A higher proportion of male cancer deaths were <65y compared with female (52% v 43%, χ^2^ 13.7, p<0.001).

Among 1,410 cancer deaths assessed for trends over time (excluding Karemo), the annual number of cancer deaths rose 41% from 150 to 212 deaths between 2003 and 2010 (Table S2, Figure S2). This increased the relative contribution of cancers to all NCD deaths from 26% to 45% (χ^2^ LT 56; p<0.001). This was due to a sharp rise in deaths attributed to neoplasms among persons ≥65y rising nearly 3-fold, and more than doubling the overall contribution of cancers to NCD deaths among older adults by 2010 (χ^2^ LT 52.9, p<0.001). Among deaths in adults aged <65y, the number of cancer deaths ascribed fell slightly over time, but due to steep reductions in other NCD deaths in adults aged <65y, the relative proportion of cancers against all NCDs increased in this younger age group, most notably among females <65y. The interVA-4 computer algorithm allocated cancers into five broad neoplasms: digestive, respiratory, male and female reproductive, oral, female breast, and other or unspecified.

- Digestive neoplasms (501 deaths): This included carcinomas of the oesophagus, gut, and liver and constituted 29% of all attributed cancers. The median age at death for digestive neoplasms was 62y (IQR 49-75), with 55% of deaths occurring among adults <65y of age. While the overall number and proportion of deaths from neoplasms classified as digestive did not change over time, there was a decrease in deaths in adults <65y dying from digestive neoplasms, with a concomitant increase among persons who died aged ≥65y.
- Respiratory/intrathoracic neoplasms (460 deaths): This includes carcinomas of the trachea, bronchus and lung; thymus; heart, mediastinum and pleura, and other/ill-defined respiratory and intrathoracic sites. These represented 27% of all fatal cancers. The median age at death for respiratory/intrathoracic neoplasms was 70y (IQR 53-79), with 39% of deaths occurring in persons <65y of age. The annual number of deaths ascribed to respiratory cancers rose from 5% to 50% of all attributed cancers. In adults aged <65y, both the proportion and the absolute number of deaths from respiratory/intrathoracic neoplasms rose significantly (χ^2^ LT: males 57.1, p<0.001; females 42.8, p<0.001). Significant linear trends were also observed among both sexes for such deaths in persons aged ≥65y.
- Reproductive neoplasms (97 deaths): In females, these include vulva, vagina, cervix uteri, corpus uteri, uterus, ovary, and other/unspecified genital sites. In males this category includes penis, prostate, testis, and other/unspecified genital sites. These neoplasms contributed 6% of all fatal cancers, 2% of male cancer deaths and 10% of female deaths. The median age at death for reproductive neoplasms was 65y (IQR 44-76), with 50% (31% male, 53% female) <65y of age.
- Female breast neoplasm (20 deaths): This was recorded as the cause of 2% of female cancer deaths with no reported deaths among males. The median age at death for female breast cancer was young - 41y (IQR 31-47); 85% of female breast cancer deaths were reported among females <65y of age.
- Oral neoplasms (33 deaths): This category includes neoplasms of the tongue, gum, floor of the mouth, palate and other/unspecified sites of the mouth. These contributed 2% of all cancers, 2% for each sex. The median age at death for oral neoplasms was 47y (IQR 34-71) with 67% (63% of male, 71% of female) of deaths occurring among persons <65y of age.
- Other or unspecified neoplasms (591 deaths): This large group aggregates a broad range of sub-categories including Kaposi sarcomas, neoplasms of the urinary tract, brain, thyroid and other endocrine glands. These were reported in 35% of all cancers. The median age at death for other/unspecified neoplasms was 67y (IQR 48-78), with 46% below 65y of age. Males died at a younger age than females with a median age of 57y (IQR 39-74), compared with 73y (IQR 60-81), respectively. The proportion and absolute number of deaths attributed to these neoplasms reduced over time, for both sexes, and among adults who died above and below 65y of age.

#### Cardio-vascular diseases

Ischemic heart disease, stroke, and other, unspecified cardiac and vascular disease (all area total of 1,384 deaths; 595 [43%] male, 798 female). The median age of death for CVDs was 73y (IQR 59-81; Figure 5), with one third of CVD-attributed deaths among adults <65y of age. Males were more likely than females to die from CVDs before the age of 65y (39% v 31%, respectively, χ^2^ 9.4, p = 0.002).

Among 1,170 CVD deaths assessed for trends over time (excluding Karemo), deaths fell marginally from 141 to 127 between 2003 and 2010 apart from a spike in deaths in 2008 (Table S2, Figure S3). This was in parallel with the overall fall in deaths from NCD causes, resulting in a steady relative contribution of CVDs to a quarter of all NCD deaths (2003–25%; 2010–27%).

- Ischemic heart disease (100 deaths): This category is limited to angina pectoris, myocardial infarction and, acute and chronic ischemic heart disease. These constituted 7% of all CVD deaths, 9% of all male and 6% of all female CVD deaths. The median age at death for was 69y (IQR 55-78) with 36% aged <65y old. The number of deaths attributed to these diseases did not increase over time.
- Stroke (327 deaths): This includes cerebral haemorrhage, occlusions, infarctions and other cerebrovascular disease and sequelae. These represented 24% of all CVD deaths, 24% for male and for female CVD deaths. The median age at death for stroke was 70y (IQR 56-79) with 42% of deaths in adults aged <65y. The number of deaths attributed to stroke did not increase over time.
- Other/unspecified cardiac and vascular diseases (957 deaths): This category comprises a broad range of circulatory and hypertensive disorders. These represent 69% of all CVD deaths. The median age at death for other/unspecified cardiac and vascular disease was 74y (IQR 61-81) with 32% of deaths occurring among adults <65y. Overall, the absolute number of deaths from these diseases varied over time, with a spike in 2008. There was a significant increase in adults ≥65y (χ^2^ LT: males 16.2, p<0.001; females 14.7, p<0.001).

#### Abdominal diseases

Acute abdomen or liver cirrhosis (all area total of 760 deaths; 411 [54%] male, 349 female). The median age of death for abdominal disease was 61y (IQR 38-76; Figure 5). Over half (55%) of deaths were in adults aged <65y of age at death.

Among 680 deaths attributed to abdominal disease assessed for trends over time (excluding Karemo), deaths fell 4-fold over time; from 113 reported in 2003, to 32 deaths in 2010 (Table S3, Figure S4). This decreased the relative contribution of deaths from abdominal diseases to all NCD deaths from 20% to 7% (χ^2^ LT 78, p<0.001).

- Acute abdomen (635 deaths): This was recorded in 84% of deaths from abdominal disease, equally for males and females. The median age at death for abdominal disease was 58y (IQR 35-75), with 59% occurring among adults aged <65y. The absolute number of deaths ascribed to acute abdomen decreased among younger aged adults over time, particularly among females.
- Liver cirrhosis (125 deaths): This includes alcoholic liver disease, toxic liver disease, hepatic failure and chronic hepatitis, not elsewhere classified, liver fibrosis/cirrhosis, inflammatory or other liver diseases. These represented 16% of deaths from abdominal disease, with a similar proportion in both sexes. The median age at death for liver cirrhosis was 71y (IQR 56-81) with 37% of adults <65y of age. The number of deaths attributed to liver cirrhosis also fell over time.

#### Pulmonary disease

Asthma, chronic or obstructive pulmonary disease (all area total of 231 deaths; 111 [48%] male, 121 female). The median age of death for pulmonary disease was 75y (IQR 59-82; Figure 5). Less than a third (31%) of deaths were among adults aged <65y.

Among 167 deaths attributed to pulmonary disease assessed for trends over time (excluding Karemo), deaths rose from 4 in 2003 to 36 in 2010 (Table S4, Figure S6). The contribution of pulmonary disease deaths to NCD deaths thus increased from <1% to 8% (χ^2^ LT 66, p<0.001).

- Asthma was contributed 39% of pulmonary disease deaths, with a median age at death of 64y (IQR 42-78) and half of deaths occurring among adults aged <65y.
- Other chronic or obstructive pulmonary disease included bronchitis, emphysema, and other chronic obstructive pulmonary disease contributing 61% of the pulmonary disease deaths, with a similar proportion in both sexes. The median age at death was 78y (IQR 70-85) with 18% occurring in adults aged <65y. Significantly fewer males were <65y (10%) compared with females (27%; p = 0.01).

#### Diseases of the metabolism

Includes diabetes mellitus, severe anaemia, and severe malnutrition (all area total of 216 deaths; 123 [57%] male, 93 female). The median age of death for diseases of the metabolism was 70y (IQR 57-79; Figure 5). Over one third (39%) of deaths attributed to metabolic disorders were among person aged <65y.

Among 198 deaths attributed to metabolic causes assessed for trends over time (excluding Karemo), deaths fell from 6% to 2% of NCD deaths between 2003 and 2010 (Table S3, Figure S5).

- Diabetes mellitus (136 deaths): This contributed 63% of deaths attributed to metabolic causes; 74% of metabolic deaths among males and 48% among females. The median age at death for diabetes mellitus was 72y (IQR 61-79), with 33% of deaths among adults aged below 65y. The number of deaths attributed to diabetes was sporadic over time.
- Severe malnutrition (39 deaths): This contributed 18% of deaths attributed to metabolic causes. The median age at death for severe malnutrition was 63y (IQR 43-79). While 93% of male malnutrition deaths died <65y of age, among females this was distributed across the age range.
- Severe anaemia (41 deaths): 19% of deaths attributed to metabolic causes were ascribed to severe anaemia. The median age at death for severe anaemia was 68y (IQR 51-79), with 39% <65y of age.

#### Renal disease

Acute kidney failure, chronic kidney disease, unspecified kidney failure (all area total of 118 deaths; 73 [62%] male, 45 female). The median age of death for renal disease was 72y (IQR 64-83; Figure 5); 28% of deaths were adults <65y of age, and was similar between sexes.

Among 108 deaths attributed to renal disease assessed for trends over time (excluding Karemo), deaths fell from 4% to 2% of NCD deaths (Table S4, Figure S7).

#### Epilepsy

(All area total of 32 deaths; 21 [66%] male, 11 female.) The median age of death attributed to epilepsy was 24y (IQR 19-35; Figure 5). Nearly all (97%) deaths were among adults <65y of age. Deaths attributed to epilepsy were constant over time, contributing ∼<1% of NCD deaths (Table S4, Figure S8).

#### Other/unspecified NCDs

Includes disorders of prostate, mental and behavioural, and degenerative musculoskeletal connective disorders (all area total of 404 deaths; 188 [47%] male, 216 female). The median age of death for all other/unspecified NCDs was 70y (IQR 47-80; Figure 5). Just under half (45%) of deaths were among adults aged <65y. The age difference by sex was significant with a median age among males of 59y (IQR 40-77), compared with females of 73y (IQR 50-82).

Among 345 deaths attributed to all other and unspecified NCDs assessed for trends over time (excluding Karemo; Table S3, Figure S9), deaths fell from 12% to 6% of NCD deaths (χ^2^ LT 21.4, p<0.001).

### Annual NCD Mortality Rates

NCD mortality rates between 2003 and 2010 showed significant trends by sex, age (15y to 65y or ≥65y) threshold, and against CD mortality rates. Trends in mortality rates ascribed to the main NCD causes of death; cancers, CVDs, and abdominal diseases were examined in greater detail.

#### Overall NCD Mortality Rates

Mortality rates (MRs) varied by year, with a modest overall reduction between 2003 (713 per 100,000) and 2010 (563 per 100,000; χ^2^ LT 4.2, p = 0.04; Table 1). NCD MRs were consistently higher among males than females. While NCD MRs among males fell significantly from 800 to 597 per 100,000 (χ^2^ LT 5.8, p = 0.02), the fall from 644 to 536 per 100,000 among females was not significant (p = 0.32). These trends comprise a significant rise in NCD MRs among both males and females in persons aged ≥65y, in tandem with very significant reductions in NCD MRs in both males and females aged <65y (Table 1). NCD MRs peaked in 2008, with an approximate 25% increase above the previous and subsequent year for both sexes.

**Table 1:** NCD Mortality Rates per 100,000 adult population by sex and 65 year old age threshold by year.

		Mortality Rates					Ratio
		Male	Female	RR^male^ (95% CI)	χ^2^	p value	F/M
All	2003	800	644	1.24 (1.05–1.47)	6.4	0.011	0.8
	2004	705	584	1.21 (1.01–1.44)	4.3	0.039	0.8
	2005	731	523	1.40 (1.17–1.68)	13.4	<0.001	0.7
	2006	805	653	1.23 (1.04–1.45)	6.1	0.013	0.8
	2007	591	597	0.99 (0.82–1.19)	0.0	0.91	1.0
	2008	848	750	1.13 (0.97–1.32)	2.4	0.12	0.9
	2009	678	557	1.22 (1.02–1.45)	4.9	0.028	0.8
	2010	597	536	1.12 (0.93–1.34)	1.4	0.24	0.9
	χ^2^ LT	5.8	0.32	MHRR 1.19 (1.12–1.26)			
	p value	0.016	0.57	Summary χ^2^ 30.1; p<0.001			
<65y	2003	517	409	1.26 (1.01–1.58)	4.2	0.04	0.8
	2004	381	285	1.36 (1.03–1.74)	4.7	0.03	0.7
	2005	417	263	1.58 (1.22–2.06)	11.9	<0.001	0.6
	2006	447	285	1.57 (1.22–2.02)	12.6	<0.001	0.6
	2007	320	250	1.28 (0.97–1.69)	3.1	0.08	0.8
	2008	478	325	1.47 (1.16–1.85)	10.7	0.001	0.7
	2009	262	188	1.39 (1.02–1.90)	4.5	0.035	0.7
	2010	283	183	1.55 (1.14–2.11)	8.0	0.005	0.6
	χ^2^ LT	22.89	30.69	MHRR 1.42 (1.30–1.56)			
	p value	<0.001	<0.001	Summary χ^2^ 56; p<0.001			
≥65y	2003	3262	2407	1.35 (1.05–1.75)	5.5	0.019	0.7
	2004	3528	2802	1.26 (0.99–1.60)	3.6	0.059	0.8
	2005	3527	2368	1.49 (1.16–1.96)	10.1	0.002	0.7
	2006	4164	3356	1.24 (1.00–1.55)	3.7	0.054	0.8
	2007	3144	3069	1.02 (0.81–1.30)	0.0	0.84	1.0
	2008	4435	3867	1.15 (0.93–1.41)	1.7	0.19	0.9
	2009	4815	3282	1.47 (1.19–1.81)	12.9	<0.001	0.7
	2010	3680	3123	1.18 (0.94–1.48)	2.0	0.16	0.8
	χ^2^ LT	6.7	14	MHRR 1.26 (1.16–1.36)			
	p value	0.01	<0.001	Summary χ^2^ 30.9; p<0.001			

Note:

Mortality rates 2003–2010 exclude villages added to the study site (Karemo) 2008–2010.

LT: linear trend 2003–2010; MHRR: Mantel Haenszel Relative Risk.

#### Comparison between NCD and CD Annual Mortality Rates (MRs)

Among males, CD MRs were significantly higher than NCD MRs each year and overall (Table 2). MRs for both CDs and NCDs both reduced over time, but dropped more for CDs than for NCDs. While the CD MR for females was ∼2-fold higher than for NCDs in 2003, by 2010 rates had fallen to an equivalent level (RR 1.11, 0.93–1.32, p = 0.25, Table 3). Among older males, MRs between CDs and NCDs were not significantly different (Table 2). Among older females, MRs for NCD rose, but fell for CD over time resulting in a significantly higher MR for NCD by 2010 (Table 3). For deaths among adults aged <65y, the comparison between CD and NCD MRs over time shows sharp falls for both CDs and NCDs. Among males and females, both CD and NCD MRs fell ∼2-fold (Tables 2, 3). While the MR for NCDs was unusually high in 2008, this was not evident for CDs.

**Table 2:** Male CD and NCD Mortality Rates per 100,000 adults by 65 year old age threshold by year.

		Mortality Rates					Ratio
		CD	NCD	RR^CD^ (95% CI)	χ2	p value	NCD/CD
All	2003	1461	800	1.83 (1.57–2.12)	64	<0.001	0.5
	2004	1481	705	2.10 (1.80–2.45)	93	<0.001	0.5
	2005	1351	731	1.85 (1.58–2.15)	63	<0.001	0.5
	2006	1296	805	1.61 (1.39–1.87)	40	<0.001	0.6
	2007	1014	591	1.72 (1.45–2.04)	39.6	<0.001	0.6
	2008	908	848	1.07 (0.92–1.25)	0.76	0.38	0.9
	2009	1063	678	1.57 (1.34–1.84)	31	<0.001	0.6
	2010	777	597	1.30 (1.09–1.55)	8.6	0.003	0.8
	χ^2^ LT	129	5.8	MHRR:1.61 (1.53–1.71)			
	p value	<0.001	0.016	Summary χ^2^ 292; p<0.001			
<65y	2003	1285	517	2.48 (2.06–2.99)	98	<0.001	0.4
	2004	1327	381	3.48 (2.83–4.29)	158	<0.001	0.3
	2005	1214	417	2.91 (2.38–3.56)	120	<0.001	0.3
	2006	1052	447	2.35 (1.93–2.87)	76	<0.001	0.4
	2007	816	320	2.55 (2.03–3.20)	69	<0.001	0.4
	2008	763	478	1.59 (1.31–1.94)	21.7	<0.001	0.6
	2009	902	262	3.44 (2.71–4.37)	116	<0.001	0.3
	2010	646	283	2.28 (1.79–2.91)	47	<0.001	0.4
	χ^2^ LT	126.5	22.89	MHRR:2.57 (2.38–2.76)			
	p value	<0.001	<0.001	Summary χ^2^ 672; p<0.001			
≥65y	2003	2997	3262	0.92 (0.71–1.20)	0.39	0.53	1.1
	2004	2828	3528	0.80 (0.62–1.04)	2.7	0.1	1.2
	2005	2565	3527	0.73 (0.55–0.95)	5.4	0.02	1.4
	2006	3591	4164	0.86 (0.68–1.10)	1.46	0.23	1.2
	2007	2877	3144	0.92 (0.70–1.20)	0.41	0.52	1.1
	2008	2320	4435	0.52 (0.40–0.68)	23.3	<0.001	1.9
	2009	2665	4815	0.55 (0.43–0.71)	21.2	<0.001	1.8
	2010	2065	3680	0.56 (0.42–0.75)	15.6	<0.001	1.8
	χ^2^ LT	5.8	6.7	MHRR:0.72 (0.65–0.79)			
	p value	0.16	0.01	Summary χ^2^ 49; p<0.001			

Note:

Mortality rates 2003–2010 exclude villages added to the study site (Karemo) 2008–2010.

LT: linear trend 2003–2010; MHRR: Mantel Haenszel Relative Risk.

**Table 3:** Female CD and NCD Mortality Rates per 100,000 adults by 65 year old age threshold by year.

		Mortality Rates					Ratio
		CD	NCD	RR^CD^ (95% CI)	χ2	p value	NCD/CD
All	2003	1266	644	1.97 (1.70–2.28)	86	<0.001	0.5
	2004	1405	584	2.40 (2.07–2.79)	114	<0.001	0.4
	2005	1337	523	2.56 (2.19–2.99)	153	<0.001	0.4
	2006	1344	653	2.06 (1.78–2.37)	103	<0.001	0.5
	2007	1074	597	1.80 (1.55–2.09)	60	<0.001	0.6
	2008	886	750	1.18 (1.02–1.36)	5.09	0.024	0.8
	2009	832	557	1.49 (1.27–1.75)	24.5	<0.001	0.7
	2010	594	536	1.11 (0.93–1.32)	1.34	0.25	0.9
	χ^2^ LT	206	0.32	MHRR:1.79 (1.70–1.89)			
	p value	<0.001	0.57	Summary χ^2^ 476; p<0.001			
<65y	2003	1208	409	2.95 (2.47–3.60)	147	<0.001	0.3
	2004	1353	285	4.75 (3.86–5.86)	261	<0.001	0.2
	2005	1230	263	4.67 (3.76–5.81)	234	<0.001	0.2
	2006	1229	285	4.32 (3.50–5.33)	223	<0.001	0.2
	2007	999	250	4.00 (3.20–5.01)	172	<0.001	0.3
	2008	817	325	2.51 (2.05–3.08)	84	<0.001	0.4
	2009	685	188	3.65 (2.82–4.72)	112	<0.001	0.3
	2010	505	183	2.76 (2.11–3.62)	60	<0.001	0.4
	χ^2^ LT	221.1	30.69	MHRR:3.66 (3.39–3.95)			
	p value	<0.001	<0.001	Summary χ^2^ 1270; p<0.001			
≥65y	2003	1699	2407	0.71 (0.54–0.93)	6.16	0.013	1.4
	2004	1789	2802	0.64 (0.49–0.83)	11.5	<0.001	1.6
	2005	2101	2368	0.89 (0.69–1.14)	0.86	0.35	1.1
	2006	2185	3356	0.65 (0.51–0.82)	13	<0.001	1.5
	2007	1609	3069	0.52 (0.41–0.68)	24.9	<0.001	1.9
	2008	1388	3867	0.36 (0.28–0.47)	64.9	<0.001	2.8
	2009	1913	3282	0.58 (0.46–0.74)	19.8	<0.001	1.7
	2010	1246	3123	0.40 (0.30–0.53)	47.4	<0.001	2.5
	χ^2^ LT	5.3	14	MHRR:0.57 (0.52–0.62)			
	p value	0.02	<0.001	Summary χ^2^ 152; p<0.001			

Note:

Mortality rates 2003–2010 exclude villages added to the study site (Karemo) 2008–2010.

LT: linear trend 2003–2010; MHRR: Mantel Haenszel Relative Risk.

#### Annual Mortality Rates for top three NCD causes

Cancer MRs increased from 200 to 262 per 100,000 population (χ^2^ LT 22.1, p<0.001; Table 4); with a significant rise for both sexes. This trend reflected rising rates among those ≥65y in males and females, with the MR in 2010 over 2-fold higher than in 2003. Conversely cancer MRs among adults <65y of age declined (except in 2008). Males had significantly higher cancer MRs than females on aggregate and by age category (Table 4). Most notable among cancers was the rise in respiratory/intrathoracic cancers which rose numerically and by percentage of all cancers over time. The MRs for CVDs did not differ significantly by sex, and showed no change over time, overall, by sex, or by age category (Table 5). CVD MRs in 2008 appeared aberrant with a doubling of MRs in adults <65y, compared with 2007 and 2009. MRs for abdominal disease decreased more than 3-fold in both sexes; this was evident in both younger and older populations (Table 6). MRs were significantly higher in males overall, and by age threshold, but varied in magnitude between years. There was no evidence of a rise in the abdominal diseases MR in 2008.

**Table 4:** Cancer Mortality Rates per 100,000 population by sex by 65y age threshold by year.

		Mortality Rates						%Respiratory Ca	
		Male	Female	Total	RR^male^ (95% CI)	χ2	p value	Male	Female
All	2003	249	162	200	1.54 (1.12–2.12)	7.04	0.008	6.1	2.9
	2004	237	151	189	1.56 (1.13–2.18)	7.22	0.007	6.3	7.8
	2005	242	158	195	1.53 (1.11–2.12)	6.84	0.009	13.4	9.0
	2006	256	225	239	1.14 (0.85–1.52)	0.77	0.38	14.8	15.6
	2007	142	159	151	0.89 (0.62–1.28)	0.38	0.54	8.0	10.1
	2008	307	280	292	1.10 (0.85–1.42)	0.52	0.47	29.5	31.7
	2009	318	224	266	1.42 (1.09–1.86)	6.76	0.009	42.6	41.0
	2010	290	239	262	1.22 (0.93-1.59)	2.04	0.15	55.2	44.9
	χ^2^ LT	52.4	18.76	22.07	MHRR 1.27 (1.15–1.41)				
	p value	0.022	<0.001	<0.001	Summary χ^2^ 20.69; p<0.001				
<65	2003	196	105	145	1.87 (1.25–2.80)	9.4	0.002	6.9	2.6
	2004	144	78	107	1.85 (1.15–2.96)	6.7	0.01	2.3	3.4
	2005	157	94	123	1.68 (1.08–2.59)	5.5	0.02	4.2	5.7
	2006	154	114	133	1.35 (0.89–2.03)	2	0.15	10.4	9.3
	2007	91	110	102	0.82 (0.51–1.32)	0.6	0.42	3.4	11.9
	2008	188	144	164	1.30 (0.91–1.87)	2.1	0.15	27.4	33.3
	2009	119	66	90	1.80 (1.10–2.96)	5.6	0.018	38.5	50.0
	2010	140	81	108	1.73 (1.10–2.71)	5.8	0.016	52.2	40.6
	χ^2^ LT	2.8	0.17	2.2	MHRR 1.50 (1.29–1.74)				
	p value	0.09	0.68	0.14	Summary χ^2^ 27; p<0.001				
≥65	2003	705	587	635	1.20 (0.70–2.06)	0.45	0.5	4.2	3.4
	2004	1050	696	839	1.51 (0.95–2.40)	3.07	0.08	11.1	11.4
	2005	991	611	762	1.62 (1.00–2.62)	3.96	0.047	26.5	12.5
	2006	1207	1034	1102	1.17 (0.78–1.76)	0.55	0.46	20.0	20.8
	2007	623	505	551	1.23 (0.70–2.18)	0.52	0.47	14.3	7.4
	2008	1468	1277	1351	1.15 (0.80–1.65)	0.58	0.45	32.0	30.4
	2009	2302	1388	1737	1.66 (1.21–2.28)	9.97	0.002	44.7	37.8
	2010	1765	1394	1537	1.27 (0.90–1.78)	1.88	0.17	57.6	46.7
	χ^2^ LT	33.75	37.5	70.7	MHRR 1.35 (1.17–1.56)				
	p value	<0.001	<0.001	<0.001	Summary χ^2^ 16.4; p<0.001				

Note:

Mortality rates 2003–2010 exclude villages added to the study site (Karemo) 2008–2010.

LT: linear trend 2003–2010; MHRR: Mantel Haenszel Relative Risk.

%Respiratory Ca– the proportion of respiratory cancer deaths among all cancer deaths by year and sex.

**Table 5:** Cardio-Vascular Disease Mortality Rates per 100,000 population by sex by 65y age threshold by year.

		Mortality Rates						% other CVDs	
		Male	Female	Total	RR^male^ (95% CI)	χ2	p value	Male	Female
All	2003	191	185	188	1.03 (0.74–1.44)	0.03	0.86	46.0	59.0
	2004	168	189	180	0.89 (0.63–1.25)	0.47	0.49	48.2	53.8
	2005	165	151	157	1.10 (0.77–1.57)	0.25	0.62	50.0	56.3
	2006	163	194	180	0.84 (0.60–1.18)	1.06	0.3	66.1	56.6
	2007	139	207	177	0.67 (0.47–0.95)	5.15	0.023	71.4	68.9
	2008	255	260	258	0.98 (0.75–1.29)	0.02	0.88	83.9	87.2
	2009	169	217	195	0.78 (0.57–1.07)	2.36	0.12	80.3	86.6
	2010	155	158	157	0.98 (0.69–1.39)	0.02	0.9	73.2	67.6
	χ^2^ LT	0	1.089	0.64	MHRR 0.90 (0.80–1.01)				
	p value	0.99	0.3	0.43	Summary χ^2^ 3.17; p = 0.075				
<65	2003	91	116	105	0.79 (0.49–1.28)	1	0.33	40.7	53.5
	2004	74	59	66	1.24 (0.69–2.25)	0.5	0.47	31.8	45.5
	2005	72	43	56	1.68 (0.88–3.20)	2.6	0.11	27.3	50.0
	2006	68	61	64	1.10 (0.61–1.99)	0.1	0.74	61.9	65.2
	2007	50	47	49	1.06 (0.54–2.08)	0	0.86	50.0	55.6
	2008	118	98	107	1.20 (0.77–1.87)	0.7	0.42	79.5	84.6
	2009	58	81	71	0.71 (0.40–1.26)	1.4	0.24	89.5	81.3
	2010	64	53	58	1.20 (0.66–2.20)	0.4	0.55	61.9	76.2
	χ^2^ LT	0.52	0.91	1.4	MHRR 1.06 (0.87–1.29)				
	p value	0.47	0.34	0.24	Summary χ^2^ 0.3; p = 0.57				
≥65	2003	1058	708	851	1.49 (0.94–2.37)	2.92	0.09	50.0	65.7
	2004	991	1153	1087	0.86 (0.56–1.31)	0.49	0.48	58.8	56.9
	2005	991	917	946	1.08 (0.70–1.67)	0.12	0.73	64.7	58.3
	2006	1056	1171	1126	0.90 (0.60–1.37)	0.24	0.63	68.6	53.3
	2007	979	1347	1205	0.73 (0.48–1.09)	2.36	0.12	81.8	72.2
	2008	1586	1443	1498	1.10 (0.78–1.55)	0.29	0.59	87.0	88.5
	2009	1272	1219	1239	1.04 (0.71–1.53)	0.05	0.83	76.2	89.2
	2010	1047	930	975	1.13 (0.73–1.73)	0.3	0.59	80.0	64.0
	χ^2^ LT	1.69	3.5	5.15	MHRR 1.01 (0.87–1.17)				
	p value	0.19	0.06	0.023	Summary χ^2^ 0.01; p = 0.92				

Note:

Mortality rates 2003–2010 exclude villages added to the study site (Karemo) 2008–2010.

LT: linear trend 2003–2010; MHRR: Mantel Haenszel Relative Risk.

% other CVDs – the proportion of other/unspecified CVDs deaths among all CVDs deaths by year and sex.

**Table 6:** Abdominal Disease Mortality Rates per 100,000 population by sex by 65y age threshold by year.

		Mortality Rates						% Liver Disease	
		Male	Female	Total	RR^male^ (95% CI)	χ2	p value	Male	Female
All	2003	146	154	150	0.94 (0.65–1.37)	0.1	0.76	20.8	15.4
	2004	153	132	141	1.15 (0.79–1.69)	0.55	0.46	13.7	17.9
	2005	168	111	136	1.52 (1.02–2.23)	4.57	0.032	28.1	12.8
	2006	166	112	136	1.47 (1.00–2.16)	3.98	0.046	10.5	25.0
	2007	150	115	131	1.31 (0.89–1.92)	1.84	0.17	22.6	18.0
	2008	121	64	90	1.87 (1.17–	7.14	0.008	11.4	24.1
	2009	83	29	53	2.86 (1.49–5.48)	10.95	<0.001	10.0	15.4
	2010	47	33	40	1.40 (0.70–2.81)	0.93	0.34	5.9	6.7
	χ^2^ LT	23.91	63.03	80.9	MHRR 1.38 (1.19–1.61)				
	p value	<0.001	<0.001	<0.001	Summary χ^2^ 17.61; p<0.001				
<65	2003	98	113	106	0.87 (0.54–1.39)	0.4	0.55	13.8	7.1
	2004	87	89	88	0.98 (0.59–1.64)	0	0.94	7.7	3.0
	2005	105	73	87	1.45 (0.87–2.42)	2	0.15	28.1	11.1
	2006	100	53	74	1.87 (1.07–3.29)	5	0.03	3.2	20.0
	2007	94	55	73	1.71 (0.98–2.98)	3.6	0.06	10.0	14.3
	2008	97	35	63	2.74 (1.46–5.14)	11	0.001	12.5	28.6
	2009	40	15	26	2.60 (0.99–6.84)	4	0.04	0.0	33.3
	2010	30	15	22	2.00 (0.73–5.11)	1.9	0.17	10.0	0.0
	χ^2^ LT	2.8	55.5	58.9	MHRR 1.47 (1.20–1.80)				
	p value	0.09	<0.001	<0.001	Summary χ^2^ 13; p<0.001				
≥65	2003	558	465	503	1.20 (0.65–2.20)	0.35	0.56	31.6	30.4
	2004	729	457	567	1.59 (0.91–2.80)	2.67	0.102	20.0	39.1
	2005	729	382	519	1.91 (1.06–3.42)	4.82	0.028	28.0	15.0
	2006	785	546	640	1.44 (0.84–2.44)	1.8	0.18	19.2	28.6
	2007	682	543	597	1.26 (0.73–2.17)	0.68	0.41	39.1	20.7
	2008	352	278	306	1.27 (0.60–2.71)	0.38	0.54	8.3	20.0
	2009	515	131	278	3.92 (1.63–9.45)	10.82	0.001	17.6	0.0
	2010	209	167	183	1.25 (0.47–3.36)	0.2	0.65	0.0	11.1
	χ^2^ LT	7.94	13.99	20.65	MHRR 1.54 (1.23–1.92)				
	p value	0.005	<0.001	<0.001	Summary χ^2^ 14.02; p<0.001				

Note:

Mortality rates 2003–2010 exclude villages added to the study site (Karemo) 2008–2010.

LT: linear trend 2003–2010; MHRR: Mantel Haenszel Relative Risk.

% Liver Disease – proportion of liver disease deaths to all deaths attributed to abdominal disease by year and gender.

## Discussion

While global studies highlight the potential growing burden of NCDs in LMIC, relatively few data from LMIC have been published [13]. Our study provides insights into the mortality burden of NCDs in adults in a rural African community. We found over a third of adult deaths were attributed to NCDs. This is similar to the INDEPTH international network which attributed 34.8% of deaths, ascribed by verbal autopsy, in 21 health and demographic surveillance sites in Africa and Asia to NCD [33]. A proportionate rise in NCDs over the surveillance period (2003 to 2010) was due to a significant increase among older adults (aged ≥65y), but was accompanied by a fall in overall NCD mortality rates among persons <65y of age. The latter appears to be in parallel to HIV-associated mortality reductions. In both age groups, annual NCD mortality rates were significantly higher in males. Over a third (36%) of all NCD deaths were attributed to cancers, with a significant increase in deaths attributed to respiratory neoplasms. CVD was the second most common cause, responsible for 29% of deaths, although our data found no evidence of a rise in mortality rates in this surveillance period.

Verbal autopsy and the use of interVA-4 software have enabled a systematic classification of cause of death over time in the HDSS. These data document the important and growing contribution of NCDs to mortality in rural African communities. While CDs have historically dominated public health concerns in LMICs, data presented here show that NCDs constitute probable cause of death in close to four in every ten adult deaths. In contrast, an estimated 80% of the health budgets in sub-Saharan African countries are allocated to acute CDs [34]. While the latter includes diagnosis and treatment of communicable diseases among children, Morhason-Bello and colleagues nevertheless argue the need for a substantial increase in investment for cancer care in Africa [34]. In our study, the ratio of NCDs to CDs among adults increased significantly between 2003 and 2010. This reflects the steady reduction in deaths from HIV/AIDS [21], [26], as well as a concomitant increase in rates of death from neoplasms among persons aged ≥65y. A third of NCD deaths were among younger adults <65 years of age, often referred to as premature – and largely preventable – deaths. A 2-fold reduction of NCD deaths was observed in this younger age group, which paralleled a reduction in the annual mortality rates for CDs, and was largely due to the reduction in deaths in adults <65 years attributed to cancers. Another trend of note was the spike in NCD deaths (notably CVD) in 2008, occurring in both sexes and both age groups. The only known change in this population was election violence causing additional migration, and interruptions to health care access and services during 2008 [24]. Indeed, HIV deaths among adults were found to rise perhaps reflecting an interruption in HIV treatment availability [35]. A similar spike in deaths among children under 5 years of age was assumed to be related to antimalarial ‘stock-outs’ at local health facilities although the role of post-election violence in the stock-outs or in childhood mortality overall could not be ruled out [25]. The spike in NCDs suggests a broader influence, deserving attention.

Neoplasms were responsible for over a third (35%) of all NCD deaths; the 41% rise between 2003 and 2010 was, as noted above, due to a sharp rise in persons aged>65y. The proportion of cancers appears to be considerably higher than that reported nationally in Kenya (where cancer-related deaths account for half that compared with CVD related deaths) [36]. This may partly reflect differing risk from exposures in rural western Kenya compared with urban environments. It more likely also reflects a difference in selection as only registered causes of death are included in the Kenyan cancer registry [36], underrepresenting deaths at home or in the community, and cause attribution (i.e. registered cause of death vs. that assigned by verbal autopsy and InterVA).

Until relatively recently, cancer was poorly recognised as a public health priority in African countries, particularly in areas with a high burden of CDs such as HIV, TB and malaria. Concern has now been raised that cancers are an emerging public health problem in Africa, with 715,000 new cases and 542,000 deaths estimated in 2008 [37]. In Kenya, the government has made NCDs a national priority [38], and has developed a cancer registry [39]. This will likely have increased public awareness, although permeation into rural communities may be somewhat less than urban settlements. In 2006, around 2 354 women were diagnosed with cervical cancer and 65% of these died of the disease [39]. Accepting the many caveats surrounding our study, our data support the need for increased focus on NCDs, especially among populations now aging due to reductions in early deaths from HIV-related diseases, and common childhood illnesses. Thus, there is an urgent need for increased attention to improve cancer screening, diagnosis and care, and for the development of surveillance systems that capture cancer diagnosis and deaths [4], [6]. Advances in current information and education services for populations, particularly rural, are required and should encourage early screening and health care seeking [40]. The continued presence of infectious diseases that are causally associated with adult malignancies such as HIV, viral hepatitis and HPV among others; and the increasing life expectancies and environmental exposures make cancer an increasing problem in SSA, compounded by limited access to appropriate care which can result in low survival [10].

The fall in cancers among adults aged <65y reflected the halving of ‘other neoplasms’ (for both sexes) and a fall in digestive cancers among males. The category ‘other neoplasms’ includes Kaposi's sarcoma, which prior to the use of interVA-4, ascribed HIV as the cause of death and was therefore classified as a CD [21], but now falls within the NCDs nomenclature. With the increasing coverage of antiretrovirals, longevity associated with lower HIV (CD) mortality rates [21], [26], will influence NCD cancer deaths. Evidence from Uganda suggests a higher risk of death in HIV-infected persons compared with non-infected persons with cancer [41]. Within the study reporting period, we identified 80 persons whose deaths were attributed to NCDs who were taking antiretroviral treatment at death, 64% of these were cancer deaths; and among these, 63% were classed as ‘other neoplasms’. This is supported by the Nairobi cancer registry data, which identifies Kaposi sarcoma to be a leading cancer in males in Kenya [39].

In addition to poverty and poor access to care, and continued exposure to infectious diseases, the rising cancer burden in SSA is considered to be a consequence of increasingly westernised lifestyles [42]. Addo and colleagues have documented a higher prevalence of hypertension in urban compared with rural settings in SSA, suggesting changing lifestyle factors are responsible [43]. Global experts have estimated that trends in increased survival, combined with increasing affluence and access to and exposure to carcinogens such as cigarette smoke will contribute towards a doubling of neoplasms in LMIC/Africa in the next 20 years [37]. In our study we noted a substantial rise in respiratory/intrathoracic neoplasms, with particularly high rates in elders, including females. While rates of smoking are reportedly low in our study area [44], cultural norms increase risk in particular age-groups; for example, smoking is more common among older widowed women (who, since the introduction of ARVs now have a lower risk of death from HIV [21].) Numerous NCDs including cancers are strongly correlated with smoking and alcohol [45]–[48]. In the HDSS, while only 6% of adults reported they currently smoked and 7% drank alcohol, intensity of exposure among users was found to be very high, with 60% drinking to excess (drunkenness) on at least half of all drinking occasions [44]. To date, evaluation of risk behaviours for NCDs such as inadequate physical activity, poor diet, tobacco smoking, and alcohol consumption have been minimal, but such data can potentially inform preventive programmes. While state of the art cancer care is expensive [34], some prevention strategies, such as early screening, and policy and environmental changes that supports individual behaviour change to reduce tobacco smoking and alcohol consumption [49], as well as non-patent protected chemotherapies can be affordable [15]. The creation of a package of affordable population prevention measures for NCD risks will support decision making on the formulation of preventive strategies and policies in LMICs [11]. The national cancer control strategy, developed for Kenya, aims to cover the continuum of prevention and control, promoting cancer prevention, early detection, improvement in diagnosis and treatment, and the promotion of cancer surveillance, registration and research [38]. Following this, comprehensive management guidelines have been developed [50].

Many barriers to diagnosis and treatment remain, however, including a lack of population awareness of symptoms leading to early diagnosis, lack of knowledge by health workers, lack of chemotherapy and radiotherapy services, and a lack of suitable pain relief and palliative care [15], [34], [51]–[53]. Reduction of exposure to important pollutants, like smoke from cooking fires inside the home [46], is being addressed through research studies supported by the Global Alliance for Clean Cookstoves, including in western Kenya [54], [55]. The age of death for some neoplasms, such as female breast cancers, is young; in our study population women died at a median age of 41y (IQR 31–47), with 85% of deaths reported among females <65y. In Ethiopia, young age and advanced stage have been associated with poor outcome [56]. This compares with the median age at death of 68 years in the USA [57]. Lack of treatment may be another component influencing early death with other contributory factors resulting in more aggressive forms or accelerating death still to be identified. Cancer Research UK investigators report black ethnicity is an independent indicator of poor female breast cancer prognosis among UK women, and note young black women develop more aggressive tumours with higher recrudescence rates than white women [58]. The contribution of a higher number of pregnancy-associated tumours, identified in meta-analysis studies to be strongly associated with prognosis [59], is of relevance to LMICs. Inclusion of prevention awareness programmes in antenatal care services would further strengthen early diagnosis and treatment opportunities in this vulnerable population. Global advocacy for implementation of HPV prevention strategies has gained momentum, with demonstration projects underway [60].

Globally, deaths from CVDs are expected to rise from 16.7 m to 23.3 m between 2002 and 2030 [7]. A leading risk factor for CVDs and deaths worldwide is high blood pressure, with the highest prevalence observed in LMIC [61]. A systematic review in SSA identified hypertension, excess sodium intake, and low physical activity, as a major public health issues and called for improved detection, treatment and control [43]. Further evidence is accumulating of protease inhibitors increasing the risk of hypertension and obesity among persons under care for HIV, of substantial importance in regions of high HIV prevalence [62]. In our rural setting, CVDs accounted for one quarter of all NCDs, with no increase detected during the study period. The lack of increase in CVDs may be due to the relatively low socio-economic status of the predominantly rural population in the HDSS.

The HDSS infrastructures could be utilized to facilitate intervention studies; for example, by providing anti-hypertensives and follow-up monitoring to persons identified to be hypertensive during mass screening at census. Such sentinel sites also provide opportunities for longitudinal surveillance of key risks such as diet, smoking and alcohol consumption.

The international community is justly striving for concerted global action with leadership, prevention, treatment, international cooperation, and monitoring and accountability proposed as the five overarching priority actions needed to respond to the global crisis of NCDs [8]. NCDs were defined as a neglected global priority by member states at the United Nations General Assembly in September 2011 [8], and the framework for monitoring global progress and a set of global targets to monitor trends and assess the progress in countries for the prevention and control of NCDs were adopted at the World Health Assembly in May 2013 [63], along with a NCDs action plan [64]. Gaps in reporting of NCDs outcomes, and the accuracy, quality, standardisation of reporting on risk factors previously argued [65], remains an important consideration however [18], [66]. Improving the quality of risk factor surveillance data is required, particularly in LMICs [6]. A multi-country study in INDEPTH sites has shown that chronic NCDs risk factor surveillance is feasible in LMIC settings [67]. There is thus ample opportunity for HDSS sites to contribute towards this.

### Study limitations

We acknowledge a number of caveats when interpreting these data. First, misclassification between CDs and NCDs, and co-morbidity between diseases may contribute to the higher proportion of NCD deaths than the 28% estimated countrywide [36], although higher exposure to CDs may contribute towards a greater NCD burden. With the substantial improvements in HIV care at least two scenarios arise: (i) where deaths from HIV mistakenly attributed to NCDs reduce over time in parallel with reductions in HIV-associated mortality, and (ii) where deaths caused by NCDs genuinely reduce over time due to the reduction in co-morbidity from HIV, including Kaposi sarcomas and lymphomas, and other cancers of viral origin. The correct attribution of cause of death nevertheless remains a concern in the absence of gold standard comparisons, such as post-mortem minimally invasive autopsies (MIA), and through prospective clinical studies. Attention is thus called for to use experience gained to modify tools in use and to strengthen surveillance systems, to develop robust methodologies that will remain valuable in the years ahead. Recent evidence suggests the sensitivity and specificity of computer algorithm based diagnoses such as InterVA-4 is, nevertheless, an improvement over physician-based ascriptions [32]. We used the WHO-compliant InterVA-4 (version 4.02) probabilistic model for cause of death assignments, which has been field tested [31], and is fully compliant with the WHO 2012 VA standard and generates causes of death categorised by ICD-10 groups [68]. We note, however that InterVA-4 attribution precludes detailing specific causes within each ICD-10 category, because any VA method cannot reveal that level of detail [69]. While the overall estimates currently generated inform of general NCD trends by ICD-10 category, improved algorithms defining individual causes would be invaluable for public health programmes. A limitation of specific causes can lead to misattribution, for example, deaths caused by complications of diabetes may be attributed to the complication arising, e.g. renal failure.

Second, we note ascription of cause of death through verbal autopsy depends on the symptoms reported; symptoms of a chronic disease might have been reported but for some cases, an underlying disease such as HIV may be undiagnosed and contribute to cause of death. This is a recognised limitation of verbal autopsy [33], and supports the need for comparison against other methods such as MIA. Capture of data retrospectively has a number of limitations, including recall bias, poor diagnosis due to lack of adequate cancer specialists and lack of facilities, poor referral and a lack of education both of patient and care provider. As such, prospective ascertainment would improve the quality of surveillance should funding be available. Third, although some questions referring to premorbid clinical symptoms are included in the VA form, the questions are possibly subject to misinterpretation by family informants. Documentation of events surrounding death among the elderly may be particularly problematic, for example, if the informant is an elderly spouse or has little formal education, or because no next of kin is available and reporting depends on persons with less knowledge of the individual. Malnutrition as a cause of death among elderly may be due to underlying disease undiagnosed or miscommunicated to informant relatives. Attempts are made for timely follow-up to reduce recall bias and minimise loss of respondents through migration, however. The sharp rise in deaths attributed to neoplasms, among persons ≥65y, may partly reflect reporting bias due to perceptions of the population on the causes of death of older persons. The rise in respiratory neoplasms reported in this dataset is not mirrored in data accrued on cancers, through the Kenyan cancer registries, such as the Nairobi register [39]. In the latter, leading neoplasms recorded are reproductive cancers such as cervix, breast, and prostate, with few cases of respiratory cancer observed. Based on 2002 data breast, cervical, and prostate cancers accounted for 23.3%, 20% and 9% of all cancers registered. Such findings have informed policy with the provision of preventive and treatment guidelines [50], [70]. Use of InterVA-4 in 21 HDSS sites across Asia and Africa similarly report higher rates of respiratory cancer compared with reproductive cancers [33]. This was also true for all three sites in Kenya (Kilifi, Kisumu, and Nairobi). This may be due to a systematic bias relating to the capture of information, or may reflect cancer risk of the population under observation, which may differ from those recorded through current cancer registries. It is clearly important that further research be conducted to ascertain the true burden of cancers in both rural and urban communities, including through prospective methods, with an expansion of cancer registries to capture disease burden in rural areas.

Fourth, the significant rise in cancer mortality rates may also reflect improvement in diagnostic test availability, public perception, or of increased reporting. We have not identified any obvious systematic bias as a cause for this rise although capacity in hospitals may be improving, as we have occasional case reports documenting a confirmatory pathology diagnosis, such as squamous cell carcinoma in a person who died in the community. Thus, for example, past respiratory cancers presumptively labeled as TB may now be better detected. Despite these limitations we believe our data will contribute towards an understanding of NCDs in LMICs, and will provide a platform for studies exploring the prevalence, risk factors, and potential interventions to control the NCD burden in the years ahead.

### Conclusion

Data generated from the HDSS in rural western Kenya illustrate the important contribution of NCDs to adult deaths among rural African communities. Our data suggest mortality from NCDs are proportionately increasing compared with CD deaths, with evidence of a rise in the absolute number of deaths from cancer, and rising mortality rates. Findings presented highlight the need for improvements in prevention, screening, diagnosis, treatment and effectively delivered care of NCDs and their risk factors [4]. Documentation of risk characteristics in routine surveillance tools needs to be strengthened to support better epidemiological study and interpretation of the aetiology of NCDs in LMICs, and improve strategies for prevention of disease.

## Supporting Information

Figure S1 **Distribution of absolute number of deaths ascribed to non-communicable diseases by type, sex, age threshold, and year of death.**
(TIF)

Figure S2 **Distribution of absolute number of deaths ascribed to neoplasms by type, age threshold, sex and year of death.**
(TIF)

Figure S3 **Distribution of absolute number of deaths ascribed to cardio-vascular diseases by type, age threshold, sex and year of death.**
(TIF)

Figure S4 **Distribution of absolute number of deaths ascribed to abdominal diseases by type, age threshold, sex and year of death.**
(TIF)

Figure S5 **Distribution of absolute number of deaths ascribed to metabolic diseases by type, age threshold, sex and year of death.**
(TIF)

Figure S6 **Distribution of absolute number of deaths ascribed to pulmonary diseases by type, age threshold, sex and year of death.**
(TIF)

Figure S7 **Distribution of absolute number of deaths ascribed to renal diseases by type, age threshold, sex and year of death.**
(TIF)

Figure S8 **Distribution of absolute number of deaths ascribed to epilepsy by type, age threshold, sex and year of death.**
(TIF)

Figure S9 **Distribution of absolute number of deaths ascribed to other and unspecified non-communicable diseases by type, age threshold, sex and year of death.**
(TIF)

Table S1 **WHO Verbal Autopsy (VA) cause of death ICD equivalent codes for Non-Communicable Diseases (NCD).**
(DOCX)

Table S2 Breakdown of Non-Communicable Disease Deaths by Age Threshold, Year of Death, and Sex: All NCDs, Cancers, and CVD: absolute number of deaths in study site, excluding Karemo.* * Time trends on absolute number of deaths 2003–2010 exclude deaths from villages added to the study site (Karemo) 2008–2010.
(DOCX)

Table S3 Breakdown of Non-Communicable Disease Deaths by Age Threshold, Year of Death, and Sex: Abdominal, Other, and Metabolic diseases: absolute number of deaths in study site, excluding Karemo.* * Time trends on absolute number of deaths 2003–2010 exclude deaths from villages added to the study site (Karemo) 2008–2010.
(DOCX)

Table S4 Breakdown of Non-Communicable Disease Deaths by Age Threshold, Year of Death, and Sex: Pulmonary, Renal Diseases and Epilepsy: absolute number of deaths in study site, excluding Karemo.* * Time trends on absolute number of deaths 2003–2010 exclude deaths from villages added to the study site (Karemo) 2008–2010.
(DOCX)
