# Supplementary figures and images for: Deaths Ascribed to Non-Communicable Diseases among Rural Kenyan Adults Are Proportionately Increasing: Evidence from a Health and Demographic Surveillance System, 2003–2010
Source: PLoS One. 2014 Nov 26;9(11):e114010. doi: 10.1371/journal.pone.0114010 (PMC4245262; doi:10.1371/journal.pone.0114010)

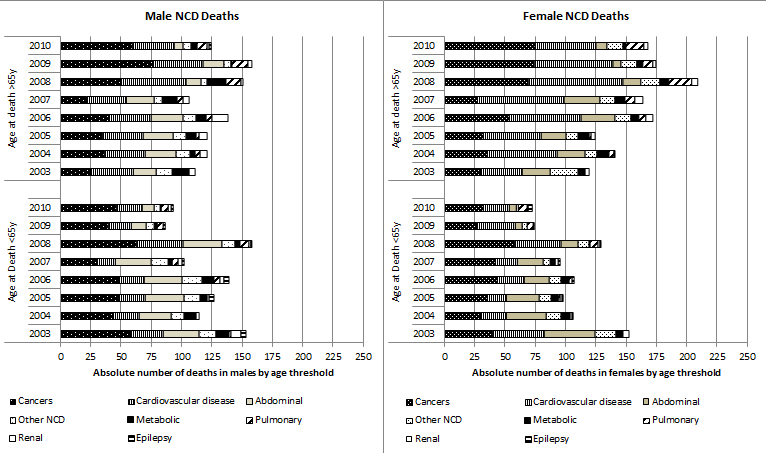

Supplement: Figure S1 — Distribution of absolute number of deaths ascribed to non-communicable diseases by type, sex, age threshold, and year of death. (TIF) [file pone.0114010.s001.tif]

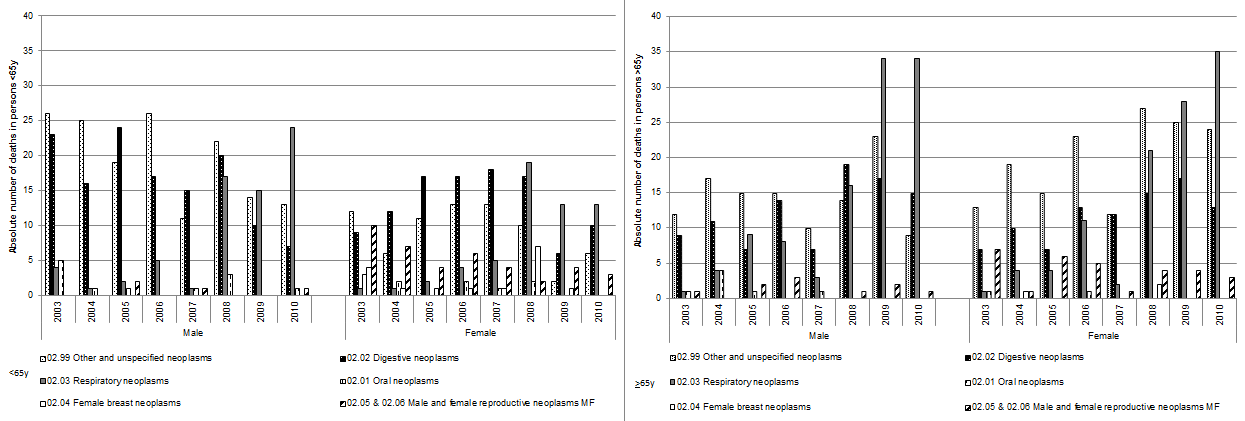

Supplement: Figure S2 — Distribution of absolute number of deaths ascribed to neoplasms by type, age threshold, sex and year of death. (TIF) [file pone.0114010.s002.tif]

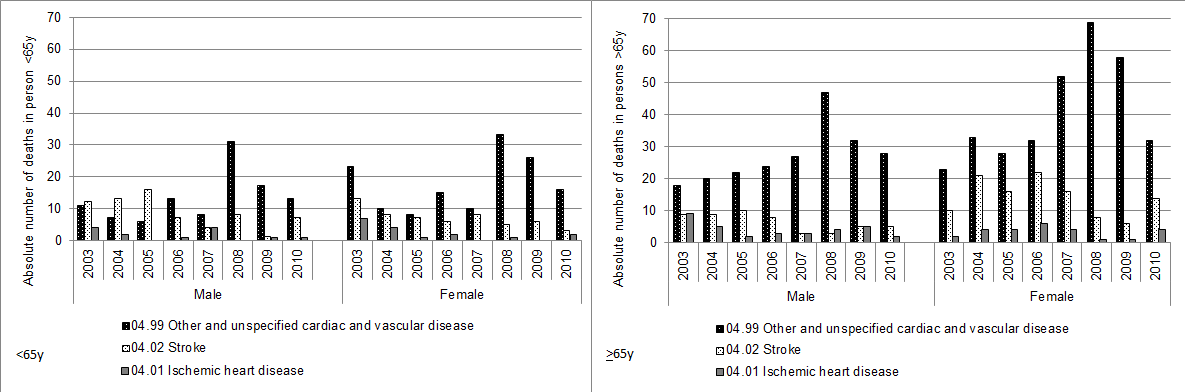

Supplement: Figure S3 — Distribution of absolute number of deaths ascribed to cardio-vascular diseases by type, age threshold, sex and year of death. (TIF) [file pone.0114010.s003.tif]

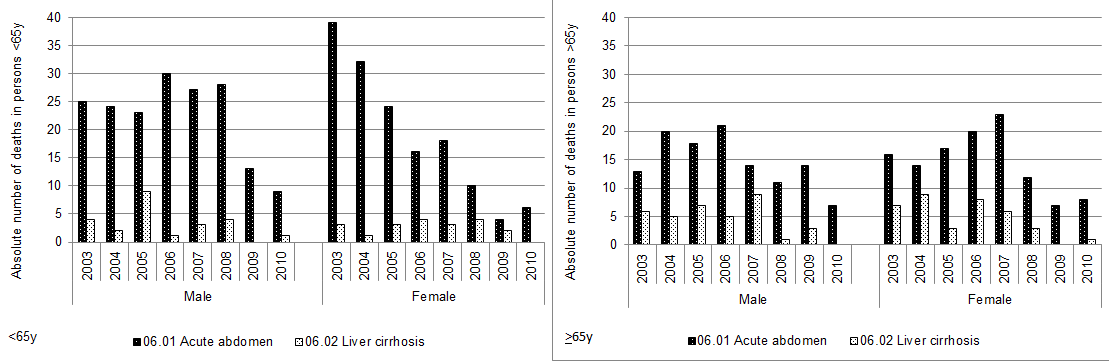

Supplement: Figure S4 — Distribution of absolute number of deaths ascribed to abdominal diseases by type, age threshold, sex and year of death. (TIF) [file pone.0114010.s004.tif]

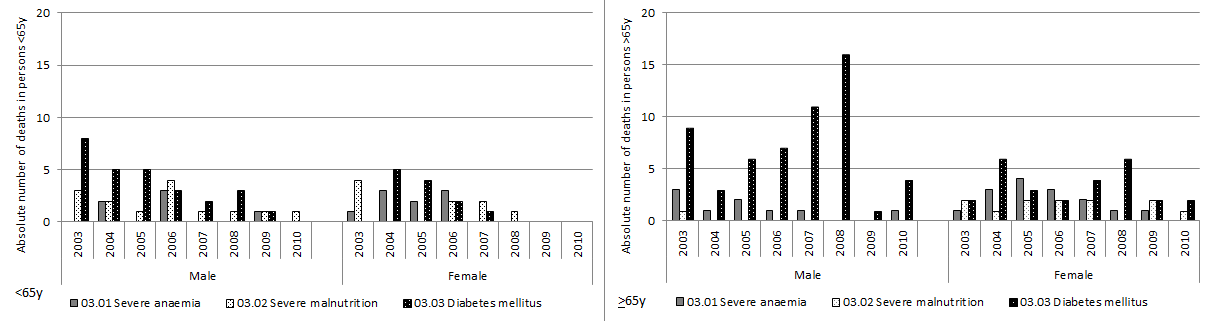

Supplement: Figure S5 — Distribution of absolute number of deaths ascribed to metabolic diseases by type, age threshold, sex and year of death. (TIF) [file pone.0114010.s005.tif]

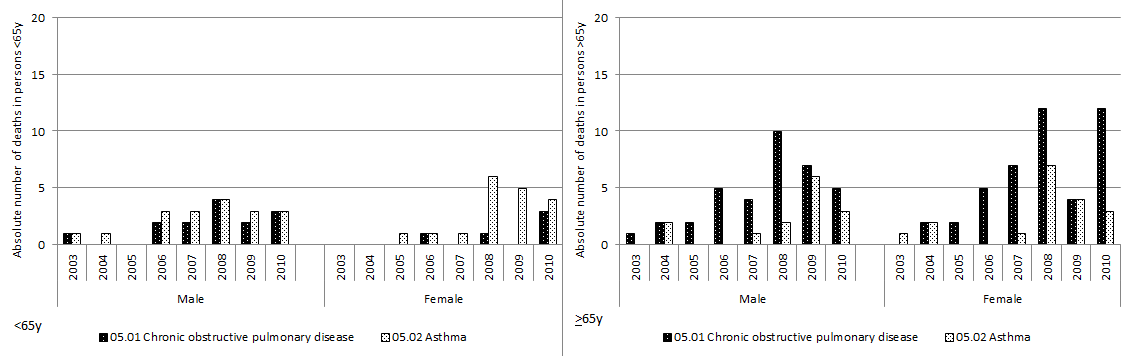

Supplement: Figure S6 — Distribution of absolute number of deaths ascribed to pulmonary diseases by type, age threshold, sex and year of death. (TIF) [file pone.0114010.s006.tif]

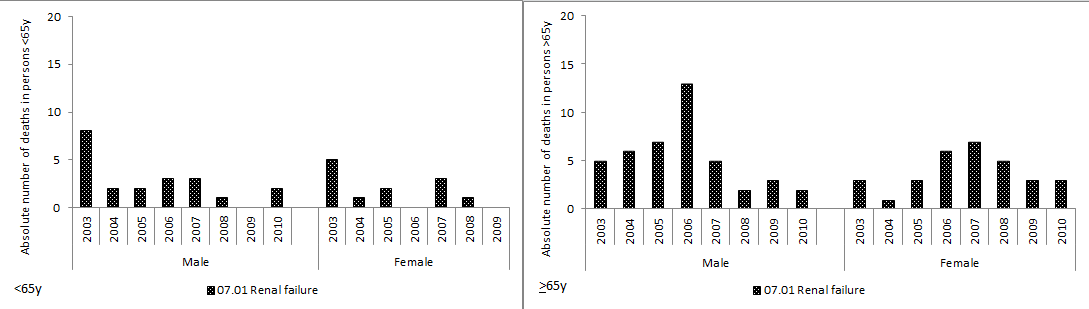

Supplement: Figure S7 — Distribution of absolute number of deaths ascribed to renal diseases by type, age threshold, sex and year of death. (TIF) [file pone.0114010.s007.tif]

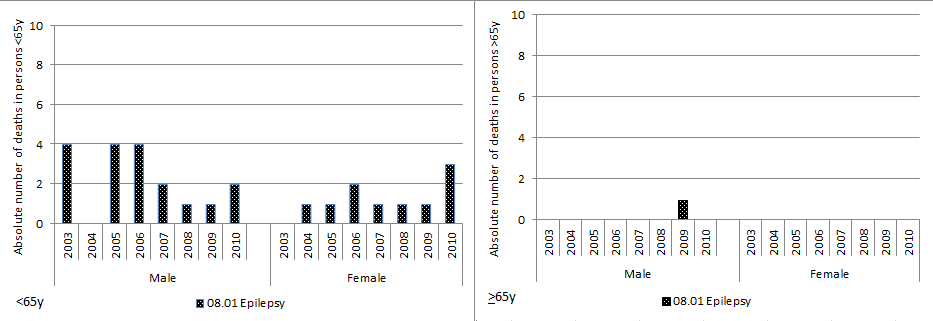

Supplement: Figure S8 — Distribution of absolute number of deaths ascribed to epilepsy by type, age threshold, sex and year of death. (TIF) [file pone.0114010.s008.tif]

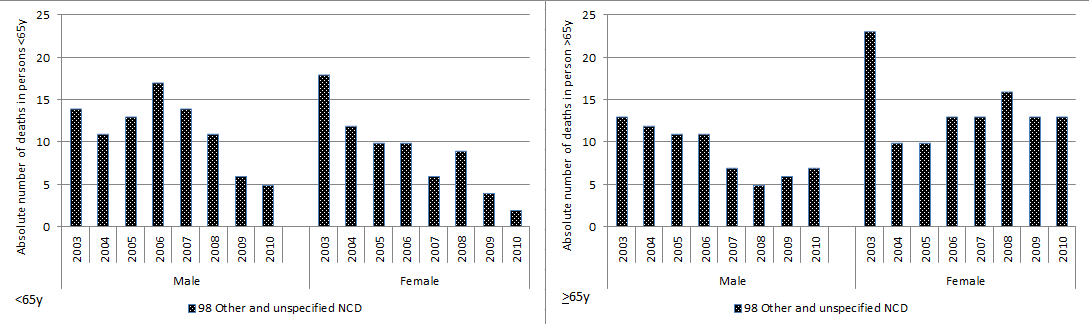

Supplement: Figure S9 — Distribution of absolute number of deaths ascribed to other and unspecified non-communicable diseases by type, age threshold, sex and year of death. (TIF) [file pone.0114010.s009.tif]
