# Supplementary material for: Deaths Ascribed to Non-Communicable Diseases among Rural Kenyan Adults Are Proportionately Increasing: Evidence from a Health and Demographic Surveillance System, 2003–2010
Source: PLoS One. 2014 Nov 26;9(11):e114010. doi: 10.1371/journal.pone.0114010 (PMC4245262; doi:10.1371/journal.pone.0114010)
Supplement: Table S1 — WHO Verbal Autopsy (VA) cause of death ICD equivalent codes for Non-Communicable Diseases (NCD). (DOCX) [file pone.0114010.s010.docx]

| Category | Sub-category | VA Code | ICD-10 Description of NCD  <http://www.icd10data.com/ICD10CM/Codes> |
| --- | --- | --- | --- |
| Neoplasms | Oral | 02.01 | C00-C06: Neoplasms of the tongue, gum, floor of mouth, palate and other/unspecified parts of mouth. |
|  | Digestive | 02.02 | C15-C26: Neoplasms of oesophagus, stomach, small intestine, colon, rectum/rectosigmoid junction, anus and canal, liver, intrahepatic bile ducts, gallbladder, other biliary tract, pancreas, other digestive. |
|  | Respiratory | 02.03 | C30-C39: Neoplasms of nasal cavity and middle ear; accessory sinuses; larynx trachea, bronchus and lung; thymus; heart, mediastinum and pleura; other/ill-defined sites in respiratory, intrathoracic organs. |
|  | Breast | 02.04 | C50: Neoplasms of the breast (female) |
|  | Reproductive  F, M | 02.05  02.06 | C51-C58: Neoplasms of vulva, vagina, cervix uteri, corpus uteri, uterus, ovary, placenta, other/unspecified female genital organs;  C60-C63: Neoplasm of penis, prostate, testis, other/unspecified male genital organs. |
|  | Other/ unspecified | 02.99 | C07-C14: Neoplasms of parotid/salivary glands, tonsil, pharynx, other sites;  C40-C49: Neoplasms of bone and cartilage; melanoma and other malignant neoplasms of the skin; mesothelioma, Kaposi's sarcoma, peripheral nerves and autonomic nervous system, retro-peritoneum and peritoneum, other connective and soft tissue;  C64-D48: Neoplasms of urinary tract; eye, brain, other parts of central nervous system; thyroid/other endocrine glands; lymphoid, hematopoietic and related tissue; unspecified/uncertain behaviour, polycythemia vera and myelodysplastic syndromes; other ill-defined, secondary, unspecified sites; malignant and secondary neuroendocrine tumours; benign and in-situ neoplasms; |
| Metabolism | Severe anaemia | 03.01 | D50-D64: Nutritional, haemolytic, aplastic and other anaemias, other bone marrow failure syndromes; |
|  | Severe malnutrition | 03.02 | E40-E46: Kwashiorkor, nutritional marasmus, marasmic kwashiorkor, protein-calorie (PCM) malnutrition, retarded development after PCM. |
|  | Diabetes mellitus | 03.03 | E10-E14: Type 1, type2, and unspecified diabetes mellitus. |
| Cardio-vascular | Acute cardiac disease | 04.01 | I20-I25: Angina pectoris, myocardial infarction and subsequent complications, acute and chronic ischemic heart diseases |
|  | Sickle cell crisis | 04.03 | D57: Sickle-cell disorders |
|  | Stroke | 04.02 | I60-I69: Intra-cerebral haemorrhage, cerebral infarction, occlusion of pre-cerebral and cerebral arteries, other cerebro-vascular diseases and sequelae. |
|  | Other/  unspecified cardiac disease | 04.99 | I00-I09: Acute rheumatic fever, chronic rheumatic heart diseases;  I10-I15: Hypertensive diseases, pulmonary heart disease;  I26-I52: Diseases of pulmonary circulation, other forms of heart disease;  I70-I99: Diseases of arteries, arterioles and capillaries; veins, lymphatic vessels and lymph nodes, not elsewhere classified; other/unspecified disorders of the circulatory system (includes emboli, thrombosis, oesophageal varices). |
| Pulmonary | Chronic obstructive pulmonary disease | 05.01 | J40-J44: Bronchitis not specified as acute or chronic, simple and muco-purulent chronic bronchitis, unspecified chronic bronchitis, emphysema, other chronic obstructive pulmonary disease. |
|  | Asthma | 05.02 | J45-J46: Asthma |
| Abdomen | Acute abdomen | 06.01 | R10: Abdominal and pelvic pain |
|  | Liver cirrhosis | 06.02 | K70-K76: Alcoholic liver disease, toxic liver disease, hepatic failure and chronic hepatitis, not elsewhere classified, fibrosis/cirrhosis of liver, inflammatory or other liver diseases. |
| Renal | Renal failure | 07.01 | N17-N19: Acute kidney failure, chronic kidney disease, unspecified kidney failure. |
| Epilepsy | Epilepsy | 08.01 | G40-G41: Epilepsy and recurrent seizures |
| Other | Other and unspecified NCD | 98 | D55-D89: Other disorders of blood and blood-forming organs, complications of procedures on spleen;  E00-E07/E15-E35: Disorders of thyroid gland; all other disorders of other endocrine glands;  E50-E90: Other nutritional deficiencies, overweight, obesity, hyper-alimentation, metabolic;  F00-F99: Mental, behavioural, neurodevelopmental disorders; inflammatory diseases of central nervous system;  G06-G37/G50-G99: Other atrophies, degenerative diseases/ disorders of nervous system including polyneuropathies;  H00-H95: Diseases of the eye, adnexa, ear and mastoid process;  J30-J39/ J47-J99: Other diseases of upper respiratory tract; bronchiectasis and all other diseases of the respiratory system;  K00-K31/K35-K38/K40-K93: Other diseases of oral cavity/salivary glands, oesophagus, stomach and duodenum; non-infective enteritis and colitis, other diseases of digestive system including appendix, hernia, liver, gallbladder, biliary tract and pancreas;  L00-L99: Diseases of the skin and subcutaneous tissue;  M00-M99: Diseases of the musculoskeletal system and connective tissue;  N00-N16/N20-N99: Other renal disease; diseases of male genital organs including prostate; benign/other disorders of breast;  R00-R09/R11-R94: Other abnormal diagnostic findings or symptoms and signs. |
