# Supplementary material for: Deaths Ascribed to Non-Communicable Diseases among Rural Kenyan Adults Are Proportionately Increasing: Evidence from a Health and Demographic Surveillance System, 2003–2010
Source: PLoS One. 2014 Nov 26;9(11):e114010. doi: 10.1371/journal.pone.0114010 (PMC4245262; doi:10.1371/journal.pone.0114010)
Supplement: Table S2 — Breakdown of Non-Communicable Disease Deaths by Age Threshold, Year of Death, and Sex: All NCDs, Cancers, and CVD: absolute number of deaths in study site, excluding Karemo.* * Time trends on absolute number of deaths 2003–2010 exclude deaths from villages added to the study site (Karemo) 2008–2010. (DOCX) [file pone.0114010.s011.docx]

|  |  | ALL NCDs | | | | Cancers | | | | Cardiovascular disease | | | |
| --- | --- | --- | --- | --- | --- | --- | --- | --- | --- | --- | --- | --- | --- |
|  |  | Males | Females | Total | % male | Males | Females | Total | % male | Males | Females | Total | % male |
| <65 | 2003 | 153 | 152 | 305 | 50 | 58 | 39 | 97 | 60 | 27 | 43 | 70 | 39 |
|  | 2004 | 114 | 106 | 220 | 52 | 43 | 29 | 72 | 60 | 22 | 22 | 44 | 50 |
|  | 2005 | 127 | 98 | 225 | 56 | 48 | 35 | 83 | 58 | 22 | 16 | 38 | 58 |
|  | 2006 | 139 | 107 | 246 | 57 | 48 | 43 | 91 | 53 | 21 | 23 | 44 | 48 |
|  | 2007 | 102 | 95 | 197 | 52 | 29 | 42 | 71 | 41 | 16 | 18 | 34 | 47 |
|  | 2008 | 158 | 129 | 287 | 55 | 62 | 57 | 119 | 52 | 39 | 39 | 78 | 50 |
|  | 2009 | 86 | 74 | 160 | 54 | 39 | 26 | 65 | 60 | 19 | 32 | 51 | 37 |
|  | 2010 | 93 | 72 | 165 | 56 | 46 | 32 | 78 | 59 | 21 | 21 | 42 | 50 |
|  | Total | 972 | 833 | 1805 | 54 | 373 | 303 | 676 | 55 | 187 | 214 | 401 | 47 |
|  |  |  |  |  |  |  |  |  |  |  |  |  |  |
| >65 | 2003 | 111 | 119 | 230 | 48 | 24 | 29 | 53 | 45 | 36 | 35 | 71 | 51 |
|  | 2004 | 121 | 141 | 262 | 46 | 36 | 35 | 71 | 51 | 34 | 58 | 92 | 37 |
|  | 2005 | 121 | 124 | 245 | 49 | 34 | 32 | 66 | 52 | 34 | 48 | 82 | 41 |
|  | 2006 | 138 | 172 | 310 | 45 | 40 | 53 | 93 | 43 | 35 | 60 | 95 | 37 |
|  | 2007 | 106 | 164 | 270 | 39 | 21 | 27 | 48 | 44 | 33 | 72 | 105 | 31 |
|  | 2008 | 151 | 209 | 360 | 42 | 50 | 69 | 119 | 42 | 54 | 78 | 132 | 41 |
|  | 2009 | 159 | 175 | 334 | 48 | 76 | 74 | 150 | 51 | 42 | 65 | 107 | 39 |
|  | 2010 | 123 | 168 | 291 | 42 | 59 | 75 | 134 | 44 | 35 | 50 | 85 | 41 |
|  | Total | 1030 | 1272 | 2302 | 45 | 340 | 394 | 734 | 46 | 303 | 466 | 769 | 39 |
|  |  |  |  |  |  |  |  |  |  |  |  |  |  |
| All | 2003 | 264 | 271 | 535 | 49 | 82 | 68 | 150 | 55 | 63 | 78 | 141 | 45 |
|  | 2004 | 235 | 247 | 482 | 49 | 79 | 64 | 143 | 55 | 56 | 80 | 136 | 41 |
|  | 2005 | 248 | 222 | 470 | 53 | 82 | 67 | 149 | 55 | 56 | 64 | 120 | 47 |
|  | 2006 | 277 | 279 | 556 | 50 | 88 | 96 | 184 | 48 | 56 | 83 | 139 | 40 |
|  | 2007 | 208 | 259 | 467 | 45 | 50 | 69 | 119 | 42 | 49 | 90 | 139 | 35 |
|  | 2008 | 309 | 338 | 647 | 48 | 112 | 126 | 238 | 47 | 93 | 117 | 210 | 44 |
|  | 2009 | 245 | 249 | 494 | 50 | 115 | 100 | 215 | 53 | 61 | 97 | 158 | 39 |
|  | 2010 | 216 | 240 | 456 | 47 | 105 | 107 | 212 | 50 | 56 | 71 | 127 | 44 |
|  | Total | 2002 | 2105 | 4107 | 49 | 713 | 697 | 1410 | 51 | 490 | 680 | 1170 | 42 |
