# Supplementary material for: Deaths Ascribed to Non-Communicable Diseases among Rural Kenyan Adults Are Proportionately Increasing: Evidence from a Health and Demographic Surveillance System, 2003–2010
Source: PLoS One. 2014 Nov 26;9(11):e114010. doi: 10.1371/journal.pone.0114010 (PMC4245262; doi:10.1371/journal.pone.0114010)
Supplement: Table S3 — Breakdown of Non-Communicable Disease Deaths by Age Threshold, Year of Death, and Sex: Abdominal, Other, and Metabolic diseases: absolute number of deaths in study site, excluding Karemo.* * Time trends on absolute number of deaths 2003–2010 exclude deaths from villages added to the study site (Karemo) 2008–2010. (DOCX) [file pone.0114010.s012.docx]

|  |  |  |  | |  | |  | |  |  | |  | |  |  | | |  | |  | |  |
| --- | --- | --- | --- | --- | --- | --- | --- | --- | --- | --- | --- | --- | --- | --- | --- | --- | --- | --- | --- | --- | --- | --- |
|  |  | Abdominal | | | | | | | Other NCD | | | | | | Metabolic | | | | | |  |  |
|  |  | Males | | Females | | Total | | % male | Males | Females | Total | | % male | | Males | Females | Total | | % male | |  |  |
| <65 | 2003 | 29 | | 42 | | 71 | | 41 | 14 | 18 | 32 | | 44 | | 11 | 5 | 16 | | 69 | |  |  |
|  | 2004 | 26 | | 33 | | 59 | | 44 | 11 | 12 | 23 | | 48 | | 9 | 8 | 17 | | 53 | |  |  |
|  | 2005 | 32 | | 27 | | 59 | | 54 | 13 | 10 | 23 | | 57 | | 6 | 6 | 12 | | 50 | |  |  |
|  | 2006 | 31 | | 20 | | 51 | | 61 | 17 | 10 | 27 | | 63 | | 10 | 7 | 17 | | 59 | |  |  |
|  | 2007 | 30 | | 21 | | 51 | | 59 | 14 | 6 | 20 | | 70 | | 3 | 3 | 6 | | 50 | |  |  |
|  | 2008 | 32 | | 14 | | 46 | | 70 | 11 | 9 | 20 | | 55 | | 4 | 1 | 5 | | 80 | |  |  |
|  | 2009 | 13 | | 6 | | 19 | | 68 | 6 | 4 | 10 | | 60 | | 3 |  | 3 | | 100 | |  |  |
|  | 2010 | 10 | | 6 | | 16 | | 63 | 5 | 2 | 7 | | 71 | | 1 |  | 1 | | 100 | |  |  |
|  | Total | 203 | | 169 | | 372 | | 55 | 91 | 71 | 162 | | 56 | | 47 | 30 | 77 | | 61 | |  |  |
|  |  |  | |  | |  | |  |  |  |  | |  | |  |  |  | |  | |  |  |
| >65 | 2003 | 19 | | 23 | | 42 | | 45 | 13 | 23 | 36 | | 36 | | 13 | 5 | 18 | | 72 | |  |  |
|  | 2004 | 25 | | 23 | | 48 | | 52 | 12 | 10 | 22 | | 55 | | 4 | 10 | 14 | | 29 | |  |  |
|  | 2005 | 25 | | 20 | | 45 | | 56 | 11 | 10 | 21 | | 52 | | 8 | 9 | 17 | | 47 | |  |  |
|  | 2006 | 26 | | 28 | | 54 | | 48 | 11 | 13 | 24 | | 46 | | 8 | 7 | 15 | | 53 | |  |  |
|  | 2007 | 23 | | 29 | | 52 | | 44 | 7 | 13 | 20 | | 35 | | 12 | 8 | 20 | | 60 | |  |  |
|  | 2008 | 12 | | 15 | | 27 | | 44 | 5 | 16 | 21 | | 24 | | 16 | 7 | 23 | | 70 | |  |  |
|  | 2009 | 17 | | 7 | | 24 | | 71 | 6 | 13 | 19 | | 32 | | 1 | 5 | 6 | | 17 | |  |  |
|  | 2010 | 7 | | 9 | | 16 | | 44 | 7 | 13 | 20 | | 35 | | 5 | 3 | 8 | | 63 | |  |  |
|  | Total | 154 | | 154 | | 308 | | 50 | 72 | 111 | 183 | | 39 | | 67 | 54 | 121 | | 55 | |  |  |
|  |  |  | |  | |  | |  |  |  |  | |  | |  |  |  | |  | |  |  |
| All | 2003 | 48 | | 65 | | 113 | | 42 | 27 | 41 | 68 | | 40 | | 24 | 10 | 34 | | 71 | |  |  |
|  | 2004 | 51 | | 56 | | 107 | | 48 | 23 | 22 | 45 | | 51 | | 13 | 18 | 31 | | 42 | |  |  |
|  | 2005 | 57 | | 47 | | 104 | | 55 | 24 | 20 | 44 | | 55 | | 14 | 15 | 29 | | 48 | |  |  |
|  | 2006 | 57 | | 48 | | 105 | | 54 | 28 | 23 | 51 | | 55 | | 18 | 14 | 32 | | 56 | |  |  |
|  | 2007 | 53 | | 50 | | 103 | | 51 | 21 | 19 | 40 | | 53 | | 15 | 11 | 26 | | 58 | |  |  |
|  | 2008 | 44 | | 29 | | 73 | | 60 | 16 | 25 | 41 | | 39 | | 20 | 8 | 28 | | 71 | |  |  |
|  | 2009 | 30 | | 13 | | 43 | | 70 | 12 | 17 | 29 | | 41 | | 4 | 5 | 9 | | 44 | |  |  |
|  | 2010 | 17 | | 15 | | 32 | | 53 | 12 | 15 | 27 | | 44 | | 6 | 3 | 9 | | 67 | |  |  |
|  | Total | 357 | | 323 | | 680 | | 53 | 163 | 182 | 345 | | 47 | | 114 | 84 | 198 | | 58 | |  |  |
