# Supplementary material for: Deaths Ascribed to Non-Communicable Diseases among Rural Kenyan Adults Are Proportionately Increasing: Evidence from a Health and Demographic Surveillance System, 2003–2010
Source: PLoS One. 2014 Nov 26;9(11):e114010. doi: 10.1371/journal.pone.0114010 (PMC4245262; doi:10.1371/journal.pone.0114010)
Supplement: Table S4 — Breakdown of Non-Communicable Disease Deaths by Age Threshold, Year of Death, and Sex: Pulmonary, Renal Diseases and Epilepsy: absolute number of deaths in study site, excluding Karemo.* * Time trends on absolute number of deaths 2003–2010 exclude deaths from villages added to the study site (Karemo) 2008–2010. (DOCX) [file pone.0114010.s013.docx]

|  |  | Pulmonary | | | | Renal | | | | Epilepsy | | | |
| --- | --- | --- | --- | --- | --- | --- | --- | --- | --- | --- | --- | --- | --- |
|  |  | Males | Females | Total | % male | Males | Females | Total | % male | Males | Females | Total | % male |
| <65 | 2003 | 2 | 0 | 2 | 100 | 8 | 5 | 13 | 62 | 4 | 0 | 4 | 100 |
|  | 2004 | 1 | 0 | 1 | 100 | 2 | 1 | 3 | 67 | 0 | 1 | 1 | 0 |
|  | 2005 | 0 | 1 | 1 | 0 | 2 | 2 | 4 | 50 | 4 | 1 | 5 | 80 |
|  | 2006 | 5 | 2 | 7 | 71 | 3 | 0 | 3 | 100 | 4 | 2 | 6 | 67 |
|  | 2007 | 5 | 1 | 6 | 83 | 3 | 3 | 6 | 50 | 2 | 1 | 3 | 67 |
|  | 2008 | 8 | 7 | 15 | 53 | 1 | 1 | 2 | 50 | 1 | 1 | 2 | 50 |
|  | 2009 | 5 | 5 | 10 | 50 | 0 | 0 | 0 |  | 1 | 1 | 2 | 50 |
|  | 2010 | 6 | 7 | 13 | 46 | 2 | 1 | 3 | 67 | 2 | 3 | 5 | 40 |
|  | Total | 32 | 23 | 55 | 58 | 21 | 13 | 34 | 62 | 18 | 10 | 28 | 64 |
|  |  |  |  |  |  |  |  |  |  |  |  |  |  |
| >65 | 2003 | 1 | 1 | 2 | 50 | 5 | 3 | 8 | 63 | 0 | 0 | 0 |  |
|  | 2004 | 4 | 4 | 8 | 50 | 6 | 1 | 7 | 86 | 0 | 0 | 0 |  |
|  | 2005 | 2 | 2 | 4 | 50 | 7 | 3 | 10 | 70 | 0 | 0 | 0 |  |
|  | 2006 | 5 | 5 | 10 | 50 | 13 | 6 | 19 | 68 | 0 | 0 | 0 |  |
|  | 2007 | 5 | 8 | 13 | 38 | 5 | 7 | 12 | 42 | 0 | 0 | 0 |  |
|  | 2008 | 12 | 19 | 31 | 39 | 2 | 5 | 7 | 29 | 0 | 0 | 0 |  |
|  | 2009 | 13 | 8 | 21 | 62 | 3 | 3 | 6 | 50 | 0 | 0 | 0 |  |
|  | 2010 | 8 | 15 | 23 | 35 | 2 | 3 | 5 | 40 | 1 | 0 | 1 | 100 |
|  | Total | 50 | 62 | 112 | 45 | 43 | 31 | 74 | 58 | 1 | 0 | 1 | 100 |
|  |  |  |  |  |  |  |  |  |  |  |  |  |  |
| All | 2003 | 3 | 1 | 4 | 75 | 13 | 8 | 21 | 62 | 4 | 0 | 4 | 100 |
|  | 2004 | 5 | 4 | 9 | 56 | 8 | 2 | 10 | 80 | 0 | 1 | 1 | 0 |
|  | 2005 | 2 | 3 | 5 | 40 | 9 | 5 | 14 | 64 | 4 | 1 | 5 | 80 |
|  | 2006 | 10 | 7 | 17 | 59 | 16 | 6 | 22 | 73 | 4 | 2 | 6 | 67 |
|  | 2007 | 10 | 9 | 19 | 53 | 8 | 10 | 18 | 44 | 2 | 1 | 3 | 67 |
|  | 2008 | 20 | 26 | 46 | 43 | 3 | 6 | 9 | 33 | 1 | 1 | 2 | 50 |
|  | 2009 | 18 | 13 | 31 | 58 | 3 | 3 | 6 | 50 | 2 | 1 | 3 | 67 |
|  | 2010 | 14 | 22 | 36 | 39 | 4 | 4 | 8 | 50 | 2 | 3 | 5 | 40 |
|  | Total | 82 | 85 | 167 | 49 | 64 | 44 | 108 | 59 | 19 | 10 | 29 | 66 |
